# Pathological Complete Response to Nab-Paclitaxel–Containing Neoadjuvant Regimens in Early Triple-Negative Breast Cancer: A Systematic Review and Meta-Analysis

**DOI:** 10.3390/jcm15187321

**Published:** 2026-09-21

**Authors:** Rasha Aziz Attia Salama, Mohamed El-Tanani, Syed Arman Rabbani, Mohamedanas Patni, Bhoomendra Bhongade, Rasha Babiker, Shakta Mani Satyam, Imran Rashid Rangraze, Adil Farooq Wali, Sirajunisa Talath, Omer Eladil Abdalla

**Affiliations:** 1 Ras Al Khaimah College of Medicine, Ras Al Khaimah Medical and Health Sciences University, Ras Al Khaimah P.O. Box 11172, United Arab Emirates; mohamedanas@rakmhsu.ac.ae (M.P.); rashababiker@rakmhsu.ac.ae (R.B.); satyam@rakmhsu.ac.ae (S.M.S.); imranrashid@rakmhsu.ac.ae (I.R.R.); omereladil@rakmhsu.ac.ae (O.E.A.); 2 Ras Al Khaimah College of Pharmacy, Ras Al Khaimah Medical and Health Sciences University, Ras Al Khaimah P.O. Box 11172, United Arab Emirates; eltanani@rakmhsu.ac.ae (M.E.-T.); arman@rakmhsu.ac.ae (S.A.R.); bhoomendra@rakmhsu.ac.ae (B.B.); farooq@rakmhsu.ac.ae (A.F.W.); sirajunisa@rakmhsu.ac.ae (S.T.)

**Keywords:** triple-negative breast cancer, nab-paclitaxel, neoadjuvant therapy, pathological complete response, immune checkpoint inhibitors, systematic review, meta-analysis

## Abstract

**Background:** Triple-negative breast cancer (TNBC) is an aggressive breast cancer subtype with limited targeted treatment options. This systematic review and meta-analysis evaluated pathological complete response (pCR) outcomes associated with nab-paclitaxel-containing neoadjuvant regimens incorporating platinum chemotherapy and/or immune checkpoint inhibitors in early-stage TNBC. **Methods:** PubMed/MEDLINE, Scopus, Google Scholar, CENTRAL, and Embase were searched for eligible publications within the review window (January 2005 through 31 March 2026). Eligible studies were randomized or prospective clinical studies of non-metastatic TNBC receiving nab-paclitaxel-containing neoadjuvant regimens with platinum and/or immune checkpoint inhibition. Randomized checkpoint-inhibitor comparisons were synthesized as risk ratios; eligible prospective non-randomized pCR datasets were synthesized using a logit random-effects model with REML variance estimation and Hartung-Knapp-Sidik-Jonkman inference. **Results:** The final evidence base comprised 21 unique eligible prospective studies. Four clinically comparable randomized checkpoint-inhibitor trials (n = 1228) yielded a pooled pCR risk ratio of 1.25 (95% CI 1.12–1.41; I^2^ = 0%). Fourteen prospective non-randomized datasets (n = 539; 320 pCR events) yielded a pooled pCR proportion of 59.8% (HKSJ 95% CI 53.8–65.6%; I^2^ = 32.7%; τ^2^ = 0.0559). **Conclusions:** Within nab-paclitaxel-containing neoadjuvant backbones, checkpoint-inhibitor addition was associated with higher pCR in randomized comparative evidence. The approximately 60% pooled pCR proportion from prospective non-randomized studies is descriptive and should not be interpreted as a cross-regimen comparison because of clinical and methodological heterogeneity.

## 1. Introduction

Triple-negative breast cancer (TNBC) is a biologically aggressive subtype of breast cancer characterized by the absence of estrogen receptor (ER) expression, progesterone receptor (PR) expression, and human epidermal growth factor 2 (HER2) overexpression or amplification [1]. Accounting for approximately 15–20% of all breast cancers, TNBC is associated with earlier recurrence, higher metastatic potential, and poorer overall survival compared with other molecular breast cancer subtypes [1,2]. Unlike hormone receptor-positive or HER2-positive breast cancers, TNBC lacks well-established targeted therapeutic options in many treatment settings, making cytotoxic chemotherapy the principal therapeutic backbone for early-stage disease [2].

Neoadjuvant chemotherapy has become a standard treatment approach in early-stage and locally advanced TNBC, offering several advantages, including tumor downstaging, improved surgical operability, and the opportunity to assess treatment responsiveness in vivo [3]. Among the available efficacy endpoints, pathological complete response (pCR) is an important early measure of treatment response and a well-established individual-level prognostic marker in TNBC. Patients who achieve pCR generally experience more favorable event-free and overall survival than those with residual disease [4]. However, this prognostic association does not establish pCR as a validated trial-level surrogate for treatment effects on event-free or overall survival. Accordingly, differences in pCR rates between treatment strategies should not be interpreted as evidence of a survival benefit without direct time-to-event evidence.

Taxane-containing chemotherapy regimens remain central to neoadjuvant TNBC management. Nab-paclitaxel (nanoparticle albumin-bound paclitaxel) represents a solvent-free taxane formulation designed to improve drug delivery while reducing hypersensitivity reactions associated with solvent-based paclitaxel [5]. Its pharmacologic profile permits steroid-free administration and may enhance intratumoral drug penetration through albumin-mediated transport mechanisms [5,6]. Several prospective clinical studies have investigated nab-paclitaxel-containing neoadjuvant regimens in TNBC, demonstrating clinically relevant pathological response rates across randomized and uncontrolled settings [7,8,9].

Treatment-intensification strategies in early TNBC have included platinum agents and immune checkpoint inhibitors. Platinum agents have been investigated as treatment-intensification strategies in TNBC, including prospective nab-paclitaxel-containing regimens and broader platinum-based neoadjuvant evidence [7,8,10]. However, within the evidence synthesized in this review, the effect of a platinum component cannot be interpreted independently of the specific chemotherapy backbone and comparator used in each trial. Accordingly, platinum-containing regimens are described according to their actual randomized or prospective treatment contrast rather than as evidence for a stand-alone platinum effect.

Researchers have also investigated immune checkpoint blockades in early TNBC in the context of an immunogenic tumor microenvironment characterized by prominent tumor-infiltrating lymphocytes (TILs) [11,12]. Higher levels of TILs have also been associated with favorable treatment response and prognosis in TNBC [11,12]. In the randomized evidence considered here, checkpoint inhibitors were administered as additions to multi-agent neoadjuvant chemotherapy; therefore, the review evaluates the effect of adding checkpoint inhibition to a defined chemotherapy backbone and does not infer efficacy of immunotherapy as an isolated treatment component [13,14,15,16,17,18,19,20,21].

Therefore, this systematic review aimed to evaluate the available evidence regarding pathological complete response outcomes associated with nab-paclitaxel-containing neoadjuvant regimens incorporating platinum agents and/or immune checkpoint inhibitors in patients with early-stage triple-negative breast cancer. By focusing specifically on nab-paclitaxel-based strategies, this review seeks to provide a clinically relevant synthesis of treatment intensification approaches in this evolving therapeutic landscape.

## 2. Methods

### 2.1. Study Design and Reporting Standards

This systematic review was conducted in accordance with the Preferred Reporting Items for Systematic Reviews and Meta-Analyses (PRISMA) 2020 guidelines [22] to evaluate pCR outcomes reported with platinum-containing and/or immune-checkpoint-inhibitor-containing nab-paclitaxel neoadjuvant regimens in patients with early-stage TNBC. The completed PRISMA 2020 checklist is provided as Supplementary File S2. The review protocol was registered in PROSPERO (Registration Number: CRD420261418350) on 8 June 2026, after completion of the literature search.

### 2.2. Search Strategy

The electronic literature search was designed to maximize sensitivity and incorporated concepts related to triple-negative breast cancer (TNBC), neoadjuvant therapy, nab-paclitaxel, and platinum agents and/or immune checkpoint inhibitors. Pathological complete response (pCR) was not included as a mandatory search concept because eligible studies could report this outcome only within the full text rather than in indexed title, abstract, or keyword fields. Search terminology included alternative terms for nab-paclitaxel and relevant platinum agents, immune checkpoint inhibitor classes, and individual agents. Eligible publications were restricted to the period from January 2005 through 31 March 2026. Five sources were searched: PubMed/MEDLINE, Scopus, CENTRAL, Embase, and Google Scholar. The searches retrieved 103 records from PubMed/MEDLINE, 117 from Scopus, 140 from CENTRAL, 564 from Embase, and 300 from Google Scholar (30 relevance-ranked pages, 10 results per page), yielding a total of 1224 source records. Database-specific search strategies, platforms/providers, execution dates, search yields, publication-cutoff handling, and record-management procedures are provided in Supplementary File S1.

### 2.3. Eligibility Criteria

Studies were considered eligible for inclusion if they met all of the following criteria: randomized controlled trials or prospective clinical studies involving adult patients with early-stage or locally advanced non-metastatic TNBC (stage I–III); evaluation of neoadjuvant treatment regimens containing nab-paclitaxel; inclusion of platinum-based chemotherapy and/or immune checkpoint inhibitors as part of the treatment strategy; reporting of pCR outcomes; and availability as full-text publications in the English language. Studies were excluded if they were retrospective observational studies, case reports, or case series; review articles, editorials, commentaries, or letters; conference abstracts lacking sufficient extractable data; studies involving metastatic breast cancer populations; studies not reporting TNBC-specific outcomes; studies not involving nab-paclitaxel-containing regimens; or duplicate publications and secondary analyses derived from previously included parent trials.

### 2.4. Study Selection

**Figure 1:**
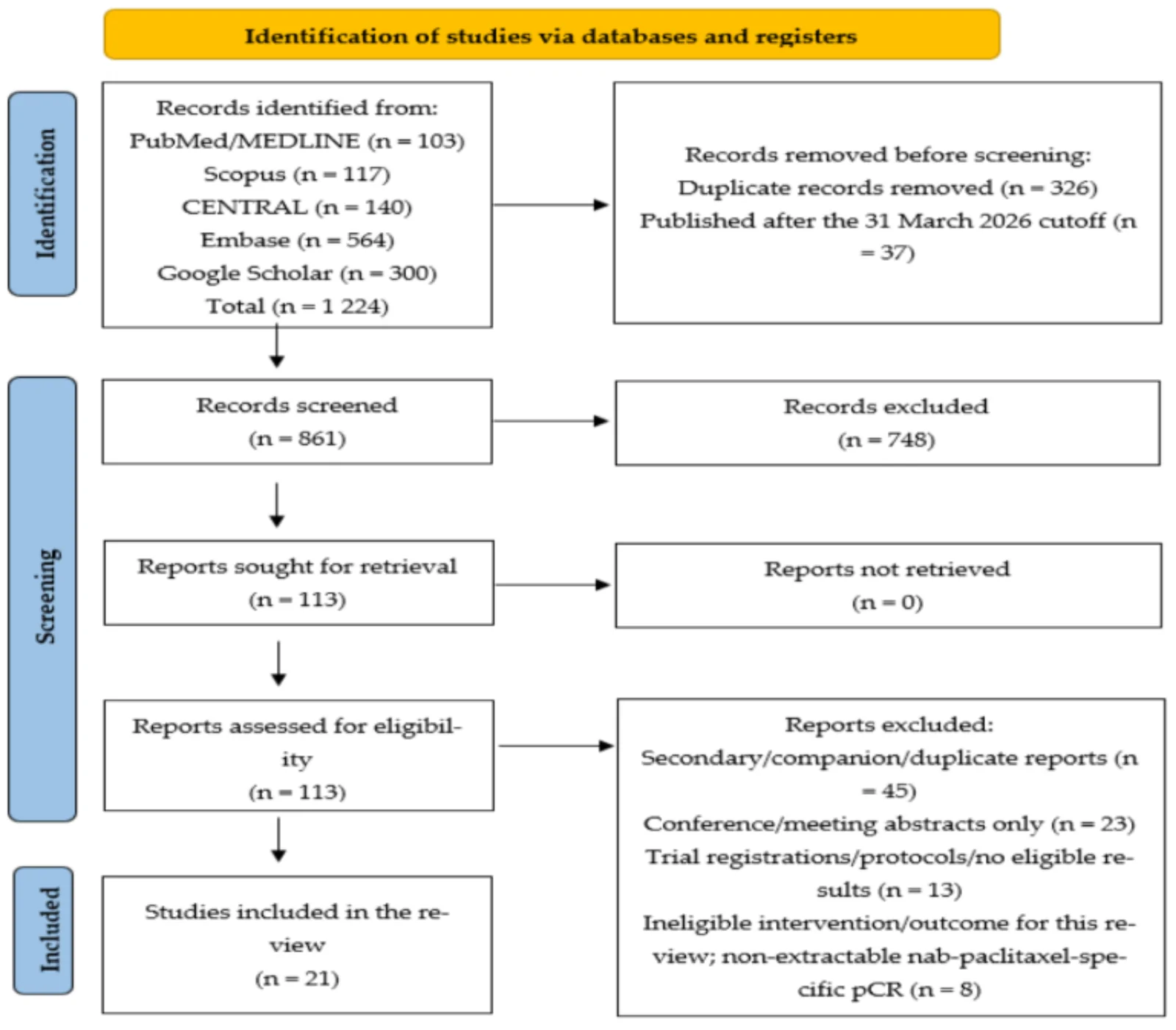
PRISMA 2020 flow diagram for study identification, screening, eligibility assessment, and inclusion.

After removal of 37 records published after the 31 March 2026 cutoff and 326 duplicate source records, 861 unique records remained for title/abstract screening. Of these, 748 were excluded, and 113 reports were sought for retrieval; all 113 reports were successfully retrieved. Accordingly, 113 full-text reports were assessed for eligibility, 92 were excluded with documented reasons, and 21 studies (21 primary reports) were included in the final review (Figure 1). The final study-family manifest of the 21 included studies is provided in Supplementary Table S1, and the complete report-level exclusion ledger for the 92 excluded reports is provided in Supplementary Table S2.

### 2.5. Data Extraction

Data were extracted independently in duplicate from the primary study reports using a standardized extraction form. For each study included in the quantitative synthesis, the reviewers extracted the pCR event count, denominator, pCR definition, treatment arm, comparator where applicable, and analytic population (e.g., intention-to-treat, randomized efficacy population, or reported subgroup). The two extraction sheets were compared item by item, and discrepancies were resolved by review of the primary report and consensus with a third reviewer. The final agreed study-level values were used for all quantitative tables, figures, and analyses. We did not use secondary reviews as the source of event counts when a primary report was available.

### 2.6. Outcome Measure

The primary outcome was pCR. The study-level pCR definition was extracted from each primary report because definitions were not completely uniform. For the descriptive prospective non-randomized synthesis, quantitative eligibility required: (1) an otherwise eligible prospective TNBC cohort receiving an eligible nab-paclitaxel-containing neoadjuvant regimen; (2) a pure pCR endpoint rather than a composite pathological response endpoint; (3) an extractable pCR numerator and denominator; and (4) a non-overlapping analytic cohort. Separately extractable TNBC subgroups were eligible when both numerator and denominator were reported. Multiple eligible non-overlapping cohorts from the same study were combined at study level where appropriate to avoid treating cohorts from one trial as independent. Randomized comparative trials were analyzed separately using risk ratios and were not incorporated into the descriptive non-randomized proportion meta-analysis. For denominator selection, the denominator used by the primary publication for its reported pCR analysis was retained (e.g., ITT/all enrolled, efficacy-evaluable, surgery-evaluable, or separately reported TNBC subgroup), without reclassifying participants whom the publication excluded from that analysis. Studies using efficacy- or surgery-evaluable denominators were then examined in a dedicated denominator sensitivity analysis. Treatment withdrawal, failure to undergo surgery, or discontinuation was counted as non-pCR only when the primary study itself included those participants in the pCR denominator as nonresponders. KEYNOTE-173 cohorts A-D were combined because they used nab-paclitaxel; cohorts E-F were excluded from quantitative synthesis because they used solvent-based paclitaxel.

### 2.7. Risk of Bias and Certainty of Evidence

Methodological quality was assessed independently by two reviewers according to study design and specifically in relation to the pCR outcome. Randomized controlled trials were evaluated using the Cochrane Risk of Bias 2 (RoB 2) tool for the effect of assignment to intervention [23]. The five RoB 2 domains—bias arising from the randomization process, bias due to deviations from intended interventions, bias due to missing outcome data, bias in measurement of the outcome, and bias in selection of the reported result—were evaluated for each randomized comparison, and an overall judgment was assigned according to the RoB 2 framework.

Prospective uncontrolled studies were assessed using the Joanna Briggs Institute (JBI) Critical Appraisal Checklist for Case Series as a design-appropriate framework for descriptive single-arm evidence [24]. The appraisal considered clarity of inclusion criteria, reliable ascertainment of the condition, participant recruitment and completeness, reporting of demographic and clinical characteristics, outcome reporting, study setting, and appropriateness of statistical analysis. These assessments were used to characterize methodological limitations of the uncontrolled evidence and were not interpreted as estimates of comparative treatment effect.

Certainty of evidence for the principal randomized comparison of checkpoint inhibitor plus chemotherapy versus chemotherapy alone was evaluated using the GRADE framework across the domains of risk of bias, inconsistency, indirectness, imprecision, and publication bias. Certainty was assessed specifically for the pCR outcome. WSG-ADAPT-TN and the randomized early-stage cohort reported by Kuemmel et al. evaluated clinically distinct treatment contrasts and were therefore considered separately rather than incorporated into the principal checkpoint-inhibitor GRADE assessment. Single-arm pooled proportions were retained as descriptive evidence and were not used to infer comparative efficacy.

### 2.8. Statistical Analysis

Comparative randomized studies were grouped according to clinical treatment contrast before synthesis. Four trials evaluating checkpoint-inhibitor addition to a corresponding chemotherapy control (IMpassion031, GeparNuevo, NeoTRIPaPDL1, and CamRelief) were pooled as risk ratios (RRs) with 95% confidence intervals from study-level pCR events and randomized denominators. The primary four-trial checkpoint-inhibitor synthesis used a DerSimonian–Laird random-effects model on the log-risk-ratio scale. WSG-ADAPT-TN and Kuemmel/CO42177 were reported separately because their randomized contrasts addressed chemotherapy/platinum strategy rather than checkpoint-inhibitor addition versus matched non-ICI control. For the descriptive prospective non-randomized synthesis, a study entered the pool when an eligible TNBC cohort receiving a nab-paclitaxel-containing regimen had an extractable pure-pCR numerator and denominator. When a publication reported a defined efficacy/surgery population, the denominator used for the study-reported primary pCR analysis was extracted and examined in sensitivity analysis. Eligible non-overlapping cohorts from the same study were combined at study-family level to avoid double counting within-study. Study proportions were logit-transformed and pooled by inverse-variance random effects using restricted maximum likelihood (REML) for τ^2^; Hartung-Knapp-Sidik-Jonkman inference was used for the primary 95% confidence interval. Heterogeneity was summarized using Cochran’s Q, I^2^, and τ^2^.

Sensitivity analyses excluded the very small Huang et al. TNBC subgroup (n = 2; 1/2 pCR), the Chen et al. radiotherapy-containing cohort (13 recruited; 10 modified-ITT efficacy-evaluable; 9/10 pCR), both together, and studies whose reported pCR denominator was an efficacy/surgery-evaluable population rather than essentially the full enrolled/treated population (TREND, Chen et al., and Zheng et al.). The same REML logit random-effects framework with Hartung-Knapp-Sidik-Jonkman inference was used for these sensitivity analyses. A DerSimonian-Laird model was used only as an estimator-sensitivity analysis for the non-randomized synthesis. Complete leave-one-out analysis was performed for the four randomized checkpoint-inhibitor trials. Because only four randomized trials contributed to that comparison, funnel-plot asymmetry tests were not used.

The primary comparative meta-analysis was restricted to the clinically coherent checkpoint-inhibitor contrast. The platinum-versus-gemcitabine comparison from WSG-ADAPT-TN was analyzed separately because it addressed a different clinical question. No omnibus meta-analysis combining these distinct randomized treatment contrasts was performed.

Because only four randomized trials contributed to the checkpoint-inhibitor meta-analysis, formal statistical assessment of small-study effects or publication bias was not performed. Robustness of the primary checkpoint-inhibitor meta-analysis was evaluated using a complete leave-one-out sensitivity analysis. Each of the four contributing randomized trials was omitted sequentially, and the pooled risk ratio (RR), 95% confidence interval (CI), and I^2^ statistic were recalculated using the same random-effects model as the primary analysis.

Meta-analytic calculations were generated from the extracted study-level event and denominator data. Randomized checkpoint-inhibitor comparisons were pooled on the log-risk-ratio scale using random-effects models, with estimator/inference robustness analyses as specified above. Prospective non-randomized pCR proportions were logit-transformed, pooled by inverse-variance random effects with REML estimation, and back-transformed. Heterogeneity was summarized using Cochran’s Q, I^2^, and τ^2^. Forest plots were generated from the final extraction dataset. Complete leave-one-out sensitivity analysis was performed for the randomized checkpoint-inhibitor synthesis, and design/denominator sensitivity analyses were performed for the prospective non-randomized synthesis.

## 3. Results

### 3.1. Study Selection

The search identified 1224 source records: PubMed/MEDLINE (n = 103), Scopus (n = 117), CENTRAL (n = 140), Embase (n = 564), and Google Scholar (n = 300). Before screening, 37 records published after the stated 31 March 2026 cutoff and 326 duplicate source records were removed, leaving 861 unique records for title/abstract screening. Of these, 748 were excluded. The remaining records yielded 113 reports sought for retrieval; all were retrieved and assessed for eligibility. Ninety-two reports were excluded at full text/report level, and 21 unique eligible studies were included.

### 3.2. Study Characteristics

The final qualitative synthesis included 21 independent prospective clinical studies, comprising randomized controlled trials, prospective phase II studies, and early-phase interventional trials evaluating nab-paclitaxel-containing neoadjuvant regimens in patients with early-stage or locally advanced non-metastatic TNBC. The included studies originated from diverse geographic regions, including Europe, North America, and Asia, reflecting broad international clinical experience. Sample sizes ranged from small early-phase studies involving approximately 30 participants to large phase III randomized trials enrolling more than 400 patients. Studies were categorized according to treatment intensification strategy (Table 1).

Platinum-containing nab-paclitaxel regimens without immune checkpoint inhibition were evaluated in WSG-ADAPT-TN, Yuan et al., and Mrózek et al. WSG-ADAPT-TN was randomized, whereas Yuan et al. and Mrózek et al. were prospective uncontrolled studies.

Immunotherapy-containing nab-paclitaxel regimens were evaluated using different chemotherapy backbones and study designs, including randomized comparisons and prospective uncontrolled studies. These findings are not used to infer the efficacy of immunotherapy as an isolated component or to rank regimens across studies.

Regimens containing both platinum and immune checkpoint inhibition were evaluated in randomized and prospective uncontrolled studies, including NeoTRIPaPDL1, CamRelief, cTRIO, and NeoSAC [15,19,20,25]. These studies differed in chemotherapy backbones, concomitant agents, populations, and comparators. Their pCR findings are therefore reported according to the actual study design and treatment contrast and are not interpreted as evidence that combined platinum–immunotherapy treatment is superior to other regimen categories or that the two components have additive effects. NeoPanDa03 additionally incorporated the VEGFR-2 inhibitor apatinib and therefore represents a multimodal regimen [21].

NeoSAC [25] evaluated sintilimab, apatinib, carboplatin, and nab-paclitaxel in a prospective single-arm design and is retained as descriptive study-specific evidence rather than comparative evidence.

### 3.3. Risk of Bias and Certainty of Evidence

Outcome-specific RoB 2 assessment was performed for the pCR outcome in all randomized studies. IMpassion031, GeparNuevo, NeoTRIPaPDL1, and CamRelief were judged to have low overall risk of bias for pCR. WSG-ADAPT-TN was judged to have some concerns because 336 patients were randomized whereas 324 constituted the reported pCR-evaluable population; this was reflected in the missing-outcome-data domain rather than assumed to be low risk. The exploratory randomized Kuemmel/CO42177 cohort was judged to have some concerns overall because of its phase Ib exploratory design and reporting context (Table 2A). The JBI appraisal was used only to describe methodological features of prospective uncontrolled studies; it was not used to determine quantitative denominator eligibility, which was adjudicated separately according to the rule in Section 2.6 (Table 2B). The GRADE assessment of the certainty of evidence for the principal randomized checkpoint-inhibitor comparison is presented in Table 2C.

### 3.4. Pathological Complete Response Outcomes

Pathological complete response outcomes are summarized according to the clinical contrast and study design (Table 3). WSG-ADAPT-TN compared nab-paclitaxel plus carboplatin with nab-paclitaxel plus gemcitabine and reported pCR rates of 45.9% and 28.7%, respectively. This finding is interpreted only as evidence for that specific chemotherapy-partner comparison and not as an estimate of an isolated platinum effect.

In the randomized checkpoint-inhibitor trials, the relevant contrast was the addition of an immune checkpoint inhibitor to a trial-specific chemotherapy control. For example, IMpassion031 reported pCR of 58% with atezolizumab plus chemotherapy versus 41% with placebo plus chemotherapy. These results support the effect of checkpoint-inhibitor addition to the studied chemotherapy backbone; they do not represent immunotherapy used independently.

Studies incorporating both platinum and checkpoint inhibition, including NeoTRIPaPDL1, CamRelief, and several prospective phase II studies, were summarized according to their individual regimen and study design. Because treatment backbones, concomitant agents, populations, and comparators differed, these findings do not permit attribution of an independent or additive effect to either platinum or immunotherapy alone.

Study-specific pCR values are presented descriptively without cross-category ranking. No formal comparison of pCR proportions across platinum-containing, immunotherapy-containing, and combined-regimen categories was performed because the underlying studies were clinically and methodologically heterogeneous; consequently, a between-category *p*-value would not provide a valid estimate of differences attributable to individual treatment components.

### 3.5. Quantitative Meta-Analysis

All 21 eligible studies were assigned an explicit analytical disposition. Four randomized checkpoint-inhibitor trials (IMpassion031, GeparNuevo, NeoTRIPaPDL1, and CamRelief) contributed to the pooled comparative RR analysis. WSG-ADAPT-TN and Kuemmel et al. were retained as randomized evidence but reported separately because their randomized contrasts addressed chemotherapy/platinum strategy rather than ICI addition versus a matched non-ICI control. Fourteen prospective non-randomized datasets with extractable pCR numerators and denominators contributed to the descriptive pooled proportion analysis. Yam et al. (atezolizumab) remained in the qualitative synthesis because the primary publication emphasized a composite pCR/RCB-I endpoint and an exact pure-pCR event count was not used in the pooled dataset.The study-level data and analytical disposition of studies contributing to the quantitative meta-analyses are summarized in Table 4.

The randomized checkpoint-inhibitor dataset comprised 1228 participants: IMpassion031 (95/165 vs. 69/168), GeparNuevo (47/88 vs. 38/86), NeoTRIPaPDL1 (67/138 vs. 63/142), and CamRelief (126/222 vs. 98/219). The pooled random-effects estimate showed a higher probability of pCR with checkpoint-inhibitor addition to the chemotherapy backbone (RR = 1.25, 95% CI 1.12–1.41; I^2^ = 0%; *p* < 0.001). WSG-ADAPT-TN was reported separately because it evaluated a chemotherapy-partner comparison rather than checkpoint-inhibitor addition.

**Figure 2:**
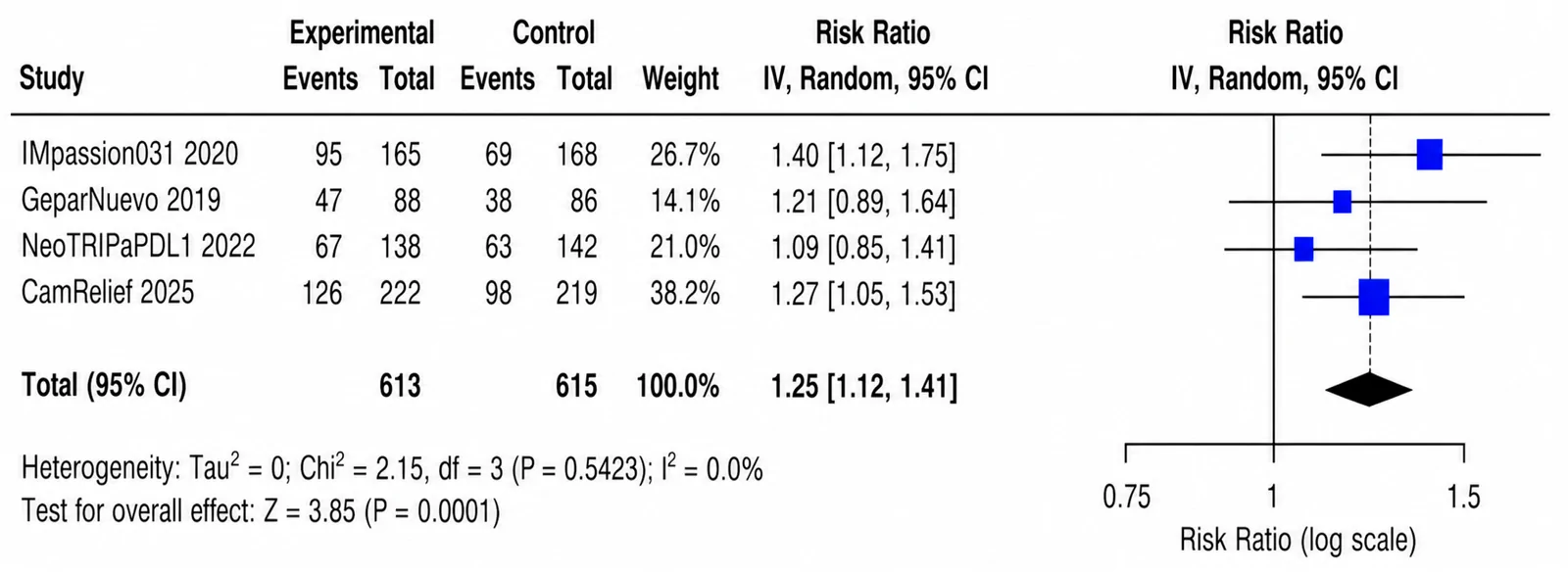
Forest plot of pathological complete response (pCR) risk ratios from four randomized checkpoint-inhibitor trials [13,14,15,20] (IMpassion031, GeparNuevo, NeoTRIPaPDL1, and CamRelief; total n = 1228) comparing checkpoint-inhibitor-containing neoadjuvant chemotherapy with the corresponding chemotherapy control. Study-specific risk ratios were calculated directly from the event counts and denominators and pooled using a DerSimonian–Laird random-effects model on the log scale. The pooled estimate was RR 1.25 (95% CI 1.12–1.41; I^2^ = 0%). WSG-ADAPT-TN and Kuemmel/CO42177 were not included in this pooled analysis because they evaluated different randomized treatment contrasts.

The comparative findings are summarized in Table 5 and displayed in Figure 2. WSG-ADAPT-TN was not included in the pooled checkpoint-inhibitor estimate because it addressed a different randomized treatment contrast.

Complete leave-one-out sensitivity analysis showed that the pooled checkpoint-inhibitor effect remained statistically significant after exclusion of each trial (Table 5). When IMpassion031 was omitted, the pooled RR was 1.20 (95% CI 1.05–1.38; I^2^ = 0%); after omission of GeparNuevo, RR = 1.26 (95% CI 1.11–1.43; I^2^ = 4.0%); after omission of NeoTRIPaPDL1, RR = 1.30 (95% CI 1.14–1.48; I^2^ = 0%); and after omission of CamRelief, RR = 1.24 (95% CI 1.07–1.45; I^2^ = 5.9%). These estimates were consistent with the primary pooled RR of 1.25 (95% CI 1.12–1.41), indicating that no single randomized trial materially altered the direction or statistical significance of the pooled result.

The prospective non-randomized pCR synthesis included 14 datasets, comprising 539 participants and 320 pCR events. Study-level pCR event counts and denominators were as follows: Yuan et al. (32/67), Mrózek et al. (6/12), NeoTENNIS (39/70), cTRIO (35/62), NeoSAC (24/34), NeoPanDa03 (23/34), KEYNOTE-173 nab-paclitaxel cohorts A–D combined (26/40), Foldi et al. (24/55), NeoImmunoboost (33/50), Wang et al. (25/39), TREND (30/44), Chen et al. (9/10), Huang et al. (1/2), and Zheng et al. (13/20). The corresponding analytic populations were ITT for NeoSAC; the phase II ITT population at the recommended durvalumab dose for Foldi et al.; efficacy-evaluable for TREND; modified ITT efficacy-evaluable for Chen et al. (10 of 13 recruited participants); the separately extractable TNBC subgroup for Huang et al.; and surgery/evaluable for Zheng et al. Study-specific denominator definitions are reported in Table 4.

Using a logit-transformed inverse-variance random-effects model with restricted maximum likelihood (REML) estimation and Hartung–Knapp–Sidik–Jonkman (HKSJ) inference, the pooled pCR proportion was 59.8% (95% CI 53.8–65.6%). Between-study heterogeneity was moderate (I^2^ = 32.7%; τ^2^ = 0.0559 on the logit scale; Cochran’s Q = 19.312, df = 13, *p* = 0.114). A sensitivity analysis using the DerSimonian–Laird estimator produced a similar pooled estimate, supporting the robustness of the result to the choice of between-study variance estimator. The REML model with HKSJ inference was retained as the primary analysis.

**Figure 3:**
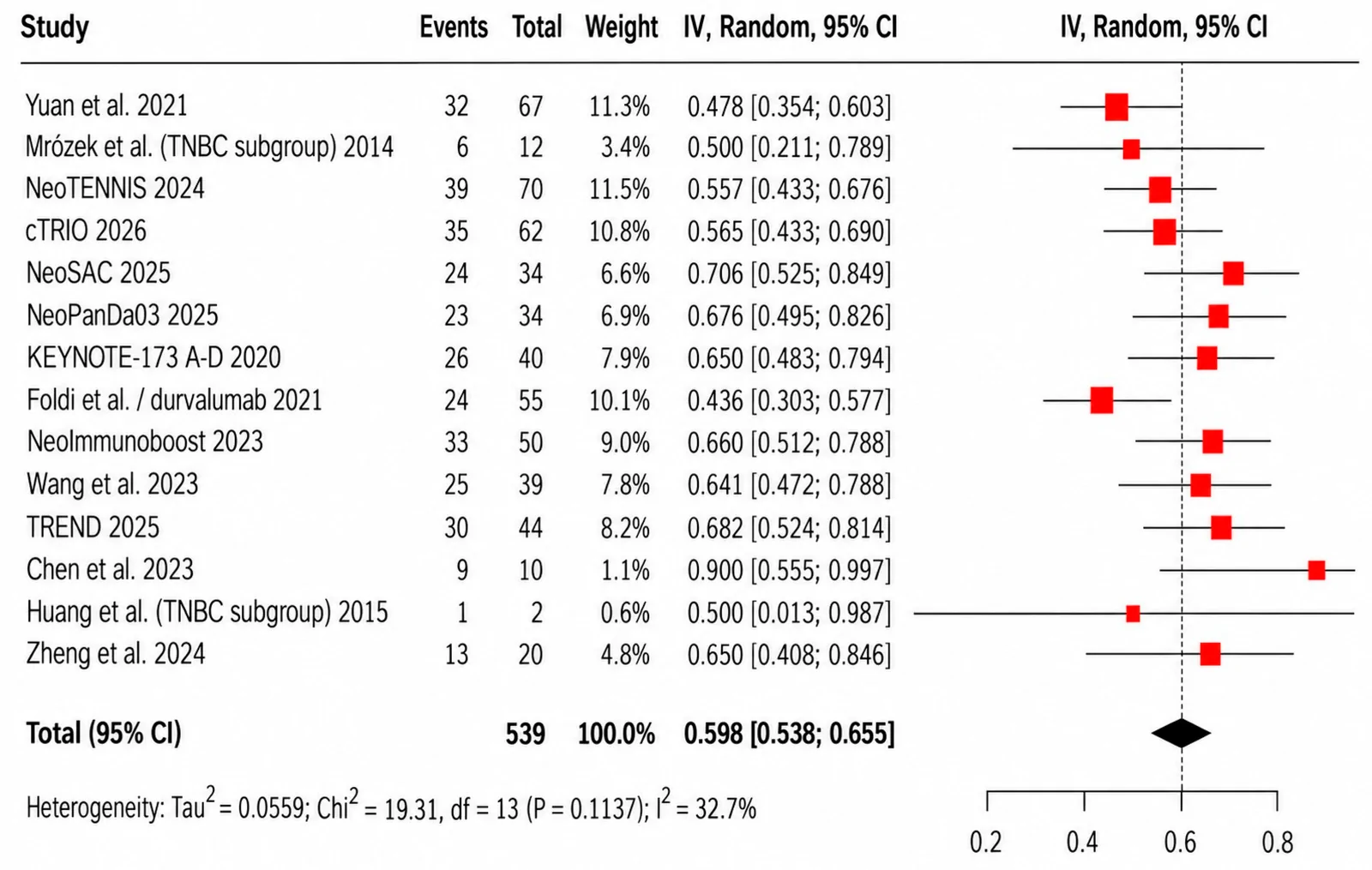
Forest plot of pCR proportions across 14 prospective non-randomized datasets [8,9,16,18,19,21,25,27,28,29,30,31,32,33] (total analysis n = 539; 320 pCR events). Study-specific proportions and 95% confidence intervals are shown. The denominators correspond to the study-specific pCR analysis populations: NeoSAC 24/34 (ITT), Foldi/durvalumab 24/55 (phase II ITT at the recommended dose), Chen 9/10 (modified-ITT efficacy-evaluable; 13 recruited), Huang 1/2 (separately extractable TNBC subgroup), TREND 30/44 (efficacy-evaluable), and Zheng 13/20 (surgery/evaluable). Proportions were logit-transformed and pooled using inverse-variance random effects with REML estimation and Hartung-Knapp-Sidik-Jonkman inference. The pooled pCR proportion was 59.8% (95% CI 53.8–65.6%), with I^2^ = 32.7% and τ^2^ = 0.0559. This synthesis is descriptive and is not intended to compare or rank treatment strategies.

No treatment-category-specific pooled proportions were used to compare the prospective non-randomized studies. Individual study-level pCR proportions are displayed in Figure 3 and the corresponding event/denominator data are provided in Table 4. These values are not ranked or interpreted as evidence of superiority or incremental benefit between treatment strategies because the underlying studies are clinically and methodologically non-equivalent.

Sensitivity analyses using the same REML logit random-effects model with Hartung-Knapp-Sidik-Jonkman inference supported the stability of the descriptive pooled estimate. Excluding the Huang et al. TNBC subgroup yielded 59.9% (95% CI 53.6–65.9%); excluding the Chen et al. cohort yielded 59.3% (95% CI 53.7–64.7%); excluding both yielded 59.4% (95% CI 53.5–65.0%). A denominator-sensitivity analysis excluding TREND, Chen et al., and Zheng et al. yielded 58.1% (95% CI 51.8–64.2%). These analyses did not materially alter the interpretation of the primary estimate.

The randomized comparative findings and the descriptive overall single-arm estimate are summarized in Table 6.

No cross-category pooled single-arm estimates are presented because the single-arm studies are not directly comparable.

Formal assessment of publication bias was not undertaken for the checkpoint-inhibitor comparative meta-analysis because only four randomized trials were available; funnel-plot asymmetry and regression-based tests are not considered reliable with such a small number of studies.

## 4. Discussion

This systematic review included 21 unique eligible prospective studies. Four randomized checkpoint-inhibitor trials involving 1228 participants yielded a pooled RR of 1.25 (95% CI 1.12–1.41; I^2^ = 0%). Separately, 14 prospective non-randomized datasets involving 539 analysis participants and 320 pCR events yielded a pooled pCR proportion of 59.8% (HKSJ 95% CI 53.8–65.6%; I^2^ = 32.7%). These analyses address different questions and should be interpreted separately; the non-randomized pooled proportion is descriptive and does not establish comparative efficacy across regimens.

TNBC remains one of the most clinically challenging breast cancer subtypes because of its aggressive biology, early recurrence pattern, and lack of endocrine or HER2-targeted treatment options [1,2]. Chemotherapy, therefore, remains a cornerstone of treatment, particularly in the neoadjuvant setting where treatment response can be directly assessed [3]. Achieving pCR has important individual-level prognostic significance in TNBC and is consistently associated with more favorable long-term outcomes, including event-free and overall survival [4]. Additional meta-analytic evidence supports the prognostic association of pCR with favorable long-term outcomes, particularly in TNBC [34,35], while residual cancer burden provides further prognostic stratification across the spectrum of residual disease after neoadjuvant therapy [36].

These patient-level associations should not be interpreted as validation of pCR as a trial-level surrogate endpoint for survival or as evidence that a treatment-induced increase in pCR necessarily produces a corresponding improvement in event-free or overall survival.

The biological rationale for platinum chemotherapy and immune checkpoint inhibition remains plausible; however, the available evidence does not permit the independent contribution of each component to be inferred across heterogeneous regimens. The randomized checkpoint-inhibitor analysis estimates the effect of adding checkpoint blockade to trial-specific chemotherapy controls, whereas WSG-ADAPT-TN addresses a carboplatin-versus-gemcitabine chemotherapy-partner comparison. The prospective single-arm evidence cannot establish comparative efficacy and is therefore interpreted only as descriptive evidence of pathological response within each study population. These findings should also be considered alongside broader platinum-based neoadjuvant evidence in TNBC, including meta-analytic data and randomized trials demonstrating increased pathological response with carboplatin-containing chemotherapy [10,37], as well as long-term follow-up from the BrighTNess trial demonstrating an event-free survival benefit with carboplatin-containing treatment [38].

Several prospective studies reported clinically notable pCR proportions with nab-paclitaxel-containing regimens, including cTRIO, NeoSAC, NeoPanDa03, and NeoTENNIS. These observations are presented as study-specific findings only. They do not demonstrate that platinum, immunotherapy, or their combination is independently effective, nor do they establish superiority or an incremental benefit attributable to one treatment component. Component-specific efficacy requires randomized designs in which the component of interest is the planned difference between otherwise comparable treatment arms.

An important distinction of this review is its specific focus on nab-paclitaxel-containing regimens. Many broader TNBC reviews combine solvent-based paclitaxel and nab-paclitaxel despite important pharmacologic differences [5,6,39]. Nab-paclitaxel avoids Cremophor-associated hypersensitivity reactions, permits steroid-free administration, and may enhance intratumoral drug delivery through albumin-mediated transport mechanisms [5,6]. These characteristics may be particularly relevant in chemoimmunotherapy combinations, where minimizing corticosteroid exposure could theoretically preserve immune activation.

The principal comparative finding of this review is limited to pCR: across four randomized trials, adding immune checkpoint blockade to the trial-specific chemotherapy backbone was associated with a higher probability of pCR. WSG-ADAPT-TN provides a separate randomized comparison of a carboplatin-containing regimen with a gemcitabine-containing regimen on a nab-paclitaxel backbone. The single-arm studies provide descriptive response estimates only and should not be used to infer superiority, treatment ranking, additive effects, or incremental benefit across regimen categories. Although pCR is prognostically important, these results should not be interpreted as demonstrating improvement in event-free survival, overall survival, toxicity, quality of life, or overall clinical benefit. Such outcomes require direct assessment from appropriately designed randomized trials and mature follow-up. Broader randomized and meta-analytic evidence in early TNBC provides relevant survival data for specific perioperative regimens [40,41]. In particular, mature follow-up from KEYNOTE-522 demonstrated an overall survival benefit with the pembrolizumab-containing perioperative regimen [42]. However, these survival data are contextual and do not represent treatment effects estimated by the present pCR meta-analysis. The broader effect of neoadjuvant checkpoint inhibition in early TNBC has been evaluated in larger syntheses; for example, Villacampa et al. included 2097 patients with TNBC and reported an increase in pCR from 46.6% to 59.9% with neoadjuvant checkpoint inhibition [41]. The specific contribution of the present review is therefore its restriction to nab-paclitaxel-containing neoadjuvant backbones and its study-level synthesis of randomized and prospective non-randomized evidence within that treatment context.

This study has several strengths. It provides a focused evaluation of nab-paclitaxel-containing neoadjuvant regimens in TNBC and incorporates contemporary platinum- and immunotherapy-based studies. The randomized evidence was synthesized according to the clinical question addressed: checkpoint-inhibitor trials formed the comparative meta-analysis, whereas the WSG-ADAPT-TN platinum comparison was analyzed separately. The structured PRISMA-based methodology, formal risk-of-bias assessment, duplicate data extraction, and quantitative pooling of clinically comparable studies support the interpretability of the evidence synthesis.

Several limitations should be acknowledged. First, only four randomized trials were suitable for the checkpoint-inhibitor comparative meta-analysis, limiting precision and prohibiting reliable formal assessment of publication bias. Second, even within this coherent comparison, treatment backbones, sequencing, and checkpoint inhibitors varied across studies. Third, randomized platinum/chemotherapy-partner evidence from WSG-ADAPT-TN and Kuemmel et al. addressed different treatment contrasts and therefore was not combined with the checkpoint-inhibitor trials. Fourth, the 14-dataset prospective non-randomized synthesis remains susceptible to selection bias, confounding, heterogeneous populations, regimens, sequencing, concomitant therapies, and pCR definitions. Some reports also used surgery- or efficacy-evaluable denominators rather than the full enrolled population; this was addressed in sensitivity analysis.

Future randomized studies should prioritize event-free survival (EFS) and overall survival (OS) alongside pCR, with adequate follow-up to determine whether improvements in pathological response translate into durable clinical benefit. Future studies should also incorporate biomarker-guided patient selection, harmonized pCR definitions, toxicity and quality-of-life endpoints, and randomized component-separation designs when the independent contribution of platinum or checkpoint inhibition is the specific question. These priorities are consistent with contemporary randomized evidence in early TNBC, in which survival benefits may emerge with longer follow-up despite earlier differences in pathological response [3,40,42].

## 5. Conclusions

The randomized evidence synthesized in this review indicates a higher probability of pCR when an immune checkpoint inhibitor is added to the chemotherapy backbones studied. WSG-ADAPT-TN separately reported higher pCR with a nab-paclitaxel plus carboplatin regimen than with a nab-paclitaxel plus gemcitabine regimen. These findings represent distinct regimen-level treatment contrasts and should not be interpreted as proving an isolated platinum effect, an isolated immunotherapy effect, additive benefit from combining treatment components, or superiority across regimen categories. The pooled proportion from prospective non-randomized studies is descriptive only. Because the present synthesis primarily evaluates pCR, no inference is made regarding event-free survival, overall survival, toxicity, quality of life, or overall clinical benefit; these outcomes require direct evidence from mature, randomized studies.

## Figures and Tables

**Table 1:** Characteristics of Included Studies by Neoadjuvant Treatment Strategy.

Study (Trial)	Year	Country	Design	Enrollment/Relevant Population (n)	Treatment Regimen	Comparator	pCR (%)	pCR Definition/Analytic Population
A. Platinum-containing chemotherapy without checkpoint inhibition								
Gluz et al. (WSG-ADAPT-TN) [7]	2018	Germany	Randomized phase II	336 enrolled; 324 pCR-evaluable	Nab-paclitaxel + carboplatin	Nab-paclitaxel + gemcitabine	45.9 vs. 28.7	ypT0/is ypN0; pCR-evaluable randomized population (324/336).
Yuan et al. [8]	2021	USA	Prospective phase II	67	Carboplatin + nab-paclitaxel	Single-arm	47.8	ypT0/Tis N0; 67 eligible evaluable patients (32/67).
Mrózek et al. [9]	2014	USA	Prospective phase II	33 enrolled; 12 TNBC subgroup	Weekly nab-paclitaxel + carboplatin + bevacizumab	Single-arm	50.0 (TNBC subgroup)	pCR in breast and axilla; TNBC subgroup n = 12 with 6 pCR events.
B. Checkpoint-inhibitor-containing regimens without a conventional platinum component								
Mittendorf et al. (IMpassion031) [13]	2020	Multinational	Phase III RCT	333 randomized (165 vs. 168)	Atezolizumab + nab-paclitaxel → AC	Placebo + chemotherapy	58.0 vs. 41.0	ypT0/is ypN0; ITT/all randomized (95/165 vs. 69/168).
Loibl et al. (GeparNuevo) [14]	2019	Germany	Randomized phase II	174 randomized (88 vs. 86)	Durvalumab + nab-paclitaxel-containing chemotherapy	Chemotherapy alone	53.4 vs. 44.2	ypT0 ypN0; randomized efficacy population (47/88 vs. 38/86).
Foldi et al. (durvalumab) [16]	2021	USA	Phase I/II single-arm	55	Durvalumab + weekly nab-paclitaxel + ddAC	None	43.6 (24/55)	Study-reported pCR; analytic population as reported in the primary study.
Yam et al. (Atezolizumab) [17]	2023	USA	Prospective phase II	33	Atezolizumab + nab-paclitaxel	Single-arm	Composite endpoint reported	Composite pathological response endpoint; qualitative only; excluded from quantitative pooling.
NeoTENNIS [18]	2024	China	Prospective phase II	70	Toripalimab + nab-paclitaxel → EC	Single-arm	55.7	tpCR = ypT0/is ypN0; ITT/all 70 enrolled (39/70); NCT04418154.
C. Platinum + checkpoint-inhibitor regimens								
Gianni et al. (NeoTRIPaPDL1) [15]	2022	Multinational	Phase III RCT	280 randomized (138 vs. 142)	Atezolizumab + carboplatin + nab-paclitaxel	Chemotherapy alone	48.6 vs. 44.4	No residual invasive disease in breast and nodes; ITT (67/138 vs. 63/142).
cTRIO [19]	2026	China	Multicenter phase II	62	Tislelizumab + nab-paclitaxel + carboplatin	Single-arm	56.5	ypT0/Tis ypN0; 62-patient analysis set (35/62).
CamRelief [20]	2025	China	Phase III RCT	441 randomized (222 vs. 219)	Camrelizumab + carboplatin + nab-paclitaxel → EC	Placebo + chemotherapy	56.8 vs. 44.7	ypT0/Tis ypN0; ITT, with no surgery counted as non-pCR (126/222 vs. 98/219).
NeoSAC [25]	2025	China	Prospective phase II	34	Nab-paclitaxel + platinum + immunotherapy	Single-arm	70.6	ypT0/Tis ypN0; ITT/all 34 enrolled (24/34).
Kuemmel et al. (tiragolumab + atezolizumab phase Ib) [26]	2025	Multinational	Open-label randomized phase Ib cohort	42 randomized (22 platinum vs. 20 non-platinum)	Tiragolumab + atezolizumab + nab-paclitaxel-containing therapy + carboplatin	Same regimen without carboplatin	45.5 vs. 55.0 (10/22 vs. 11/20)	ypT0/is ypN0; randomized early-stage cohort B; NCT04584112.
D. Immunotherapy + targeted therapy + chemotherapy								
NeoPanDa03 [21]	2025	China	Prospective phase II	34	Camrelizumab + apatinib + nab-paclitaxel-based chemotherapy	Single-arm	67.6	tpCR: ypT0/is ypN0; 34 pCR-evaluable patients (23/34).
E. Mixed immunotherapy ± platinum prospective evidence								
Schmid et al. (KEYNOTE-173) [27]	2020	Multinational	Open-label phase Ib multicohort	40 eligible in nab-paclitaxel cohorts A-D	Pembrolizumab + nab-paclitaxel; cohorts B-D also included carboplatin before anthracycline/cyclophosphamide	No randomized comparator for eligible cohort comparison	65.0 (26/40)	Study-reported pCR; eligible nab-paclitaxel cohorts A-D (26/40); descriptive only.
Fasching et al. (NeoImmunoboost) [28]	2023	Germany	Prospective phase II	50	Pembrolizumab + nab-paclitaxel-containing neoadjuvant chemotherapy	Single-arm	66.0	ypT0/is ypN0; 33/50.
Wang et al. (NCT04213898) [29]	2023	China	Prospective phase II	39	Camrelizumab + nab-paclitaxel + epirubicin	Single-arm	64.1	pCR; 25/39.
Chen et al. (NCT05132790) [30]	2023	China	Prospective pilot	13 treated; 10 efficacy-evaluable	SBRT + adebrelimab followed by adebrelimab + nab-paclitaxel + carboplatin	Single-arm	90.0	ypT0/is ypN0; 9/10 efficacy-evaluable.
Huang et al. [31]	2015	China	Prospective phase II	TNBC subgroup n = 2	Nab-paclitaxel + carboplatin	Single-arm	50.0	ypT0/is ypN0; separately extractable TNBC subgroup n = 2 (1/2); retained with tiny-subgroup sensitivity analysis.
Zheng et al. (NCT04676997) [32]	2024	China	Prospective phase II	23 treated; 20 surgery/evaluable	Camrelizumab + nab-paclitaxel-containing non-platinum chemotherapy	Single-arm	65.0	ypT0/is ypN0; 13/20 surgery/evaluable; NCT04676997.
Zhang et al. (TREND) [33]	2025	China	Prospective phase II	53 enrolled; 44 efficacy-evaluable	Tislelizumab + nab-paclitaxel followed by EC	Single-arm	68.2	pCR; 30/44 efficacy-evaluable.

Notes: Definitions of pathological complete response (pCR) varied slightly across studies but were predominantly based on ypT0/is ypN0 (absence of residual invasive disease in the breast and axillary lymph nodes). Studies reporting comparative outcomes present pCR rates for intervention versus control groups. Composite pathological response endpoints reported by individual studies were retained in the qualitative synthesis but were excluded from quantitative pooling when exact pCR event counts were unavailable. NeoPanDa03 additionally incorporated the VEGFR-2 inhibitor apatinib alongside camrelizumab and chemotherapy and therefore represents a multimodal treatment-intensification strategy rather than a conventional platinum-immunotherapy regimen. All included studies enrolled adults with early-stage or locally advanced non-metastatic TNBC (stage I-III under the review eligibility framework); however, eligibility details, chemotherapy backbones, concomitant agents, comparators, pCR definitions, and analytic populations varied across studies. These characteristics should therefore be considered when assessing comparability and applicability. Studies are grouped by the principal treatment strategy evaluated to facilitate assessment of clinical comparability. Kuemmel et al. is displayed within the platinum + checkpoint-inhibitor section but represents a distinct exploratory randomized carboplatin contrast and was not pooled with the four principal checkpoint-inhibitor RCTs. KEYNOTE-173 is shown separately because eligible nab-paclitaxel cohorts included both platinum-containing and non-platinum regimens.

**Table 2:** Risk of Bias Assessment and Certainty of Evidence for Included Studies.

**A.** pCR-Specific Cochrane RoB 2 Assessment of Randomized Studies											
**Study**		**D1 Randomization**		**D2 Deviations**		**D3 Missing Data**	**D4 pCR Measurement**		**D5 Report Selection**	**Overall**	
WSG-ADAPT-TN [7]		Low		Low		Some concerns	Low		Low	Some concerns	
IMpassion031 [13]		Low		Low		Low	Low		Low	Low	
GeparNuevo [14]		Low		Low		Low	Low		Low	Low	
NeoTRIPaPDL1 [15]		Low		Low		Low	Low		Low	Low	
CamRelief [20]		Low		Low		Low	Low		Low	Low	
Kuemmel et al. [26]		Some concerns		Low		Low	Low		Some concerns	Some concerns	
**B**. JBI Critical Appraisal of Prospective Uncontrolled Studies											
**Study**	**Q1**	**Q2**	**Q3**	**Q4**	**Q5**	**Q6**	**Q7**	**Q8**	**Q9**	**Q10**	**Appraisal**
Yuan et al. [8]	Y	Y	Y	U	Y	Y	Y	Y	Y	Y	Include
Mrózek et al. (TNBC subgroup) [9]	Y	Y	Y	U	Y	Y	Y	Y	Y	Y	Include
Foldi et al. (durvalumab) [16]	Y	Y	Y	U	Y	Y	Y	Y	Y	Y	Include
Yam et al. (atezolizumab) [17]	Y	Y	Y	U	U	Y	Y	Y	Y	Y	Include, qualitative
NeoTENNIS [18]	Y	Y	Y	U	Y	Y	Y	Y	Y	Y	Include
cTRIO [19]	Y	Y	Y	U	Y	Y	Y	Y	Y	Y	Include
NeoSAC [25]	Y	Y	Y	U	Y	Y	Y	Y	Y	Y	Include
NeoPanDa03 [21]	Y	Y	Y	U	N	Y	Y	Y	Y	Y	Include with limitation
KEYNOTE-173 cohorts A-D [27]	Y	Y	Y	U	Y	Y	Y	Y	Y	Y	Include
NeoImmunoboost [28]	Y	Y	Y	U	Y	Y	Y	U	Y	Y	Include
Wang et al. [29]	Y	Y	Y	U	Y	Y	Y	Y	Y	Y	Include
TREND [33]	Y	Y	Y	U	Y	Y	Y	Y	Y	Y	Include
Chen et al. [30]	Y	Y	Y	U	Y	Y	Y	Y	Y	Y	Include
Huang et al. TNBC subgroup [31]	Y	Y	Y	U	Y	Y	Y	Y	Y	Y	Include
Zheng et al. [32]	Y	Y	Y	U	Y	Y	Y	Y	Y	Y	Include
**C.** GRADE Summary of Findings for Checkpoint Inhibitor Plus Chemotherapy versus Chemotherapy Alone											
**Outcome**			**Studies/Participants**		**Relative Effect**		**Anticipated Absolute Effect**			**GRADE Certainty**	
Pathological Complete Response (pCR)			4 RCTs; n = 1228		RR 1.25 (95% CI 1.12–1.41)		436 per 1000 with chemotherapy alone; 545 per 1000 with checkpoint inhibitor + chemotherapy; 109 more per 1000 (52–179 more)			High	

Notes: Section A: RoB 2 judgments are outcome-specific for pCR. WSG-ADAPT-TN—randomized design and objective pathological endpoint supported low concern in D1, D2, D4, and D5, but 324/336 randomized patients comprised the reported pCR-evaluable population, yielding some concerns for D3 and overall. IMpassion031—333 randomized patients were represented in the ITT pCR comparison (95/165 vs. 69/168), supporting low concern for missing pCR data; no material concern was identified for randomization, deviations, pathological outcome measurement, or selective pCR reporting. GeparNuevo—174 randomized patients (88 vs. 86) comprised the reported randomized efficacy population, with objective pathological assessment and no material domain-level concern identified. NeoTRIPaPDL1—280 randomized patients (138 vs. 142) were analyzed for the pCR endpoint using the randomized/ITT denominator, supporting low concern across domains. CamRelief—441 randomized patients (222 vs. 219) were analyzed by ITT, and participants not undergoing surgery were counted as non-pCR. Kuemmel/CO42177—missing pCR data were not a material concern in the 42-patient randomized cohort, but the exploratory phase Ib context and reporting structure produced some concerns for D1/D5 and overall. Section B: JBI: Y = Yes; N = No; U = Unclear. The JBI appraisal characterizes reporting and methodological features of uncontrolled studies and does not override the denominator rule in Section 2.6. NeoPanDa03 excluded one enrolled participant who withdrew because of treatment intolerance from the reported pCR efficacy denominator; this denominator choice is handled in the quantitative ledger and sensitivity analysis rather than inferred from the JBI appraisal. Section C: The anticipated absolute effect and its 95% CI were calculated from the observed control-group risk (436 per 1000) and pooled RR (1.25, 95% CI 1.12–1.41). GRADE certainty was assessed across the four randomized checkpoint-inhibitor trials. Risk of bias, inconsistency, indirectness, and imprecision were not downgraded; formal assessment of small-study effects was not performed because only four RCTs were available. Overall certainty was High. Abbreviations: CI, confidence interval; GRADE, Grading of Recommendations Assessment, Development and Evaluation; ITT, intention-to-treat; JBI, Joanna Briggs Institute; pCR, pathological complete response; RCT, randomized controlled trial; RR, risk ratio; TNBC, triple-negative breast cancer.

**Table 3:** Pathological Complete Response Outcomes by Treatment Strategy.

Clinical Evidence Group	Study/Trial	Regimen	Enrollment/Relevant Population (n)	pCR (%)	Key Finding
Platinum-containing regimens	WSG-ADAPT-TN [7]	Nab-paclitaxel + carboplatin vs. nab-paclitaxel + gemcitabine	336 enrolled; 324 pCR-evaluable	45.9 vs. 28.7	Higher pCR in the carboplatin-containing arm vs. the gemcitabine-containing arm; regimen-level contrast
	Yuan et al. [8]	Carboplatin + nab-paclitaxel	67	47.8	Study-specific pCR; descriptive only
	Mrózek et al. [9]	Nab-paclitaxel + carboplatin + bevacizumab	33 enrolled; 12 TNBC subgroup	50.0	Study-specific TNBC pCR; descriptive only
Immunotherapy-containing regimens	IMpassion031 [13]	Atezolizumab + nab-paclitaxel followed by AC	333 randomized (165 vs. 168)	58.0 vs. 41.0	Higher pCR with checkpoint-inhibitor addition to matched chemotherapy
	GeparNuevo [14]	Durvalumab + nab-paclitaxel-based chemotherapy	174 randomized (88 vs. 86)	53.4 vs. 44.2	Higher pCR in the checkpoint-inhibitor arm; randomized regimen-level contrast
	Foldi et al. (durvalumab) [16]	Durvalumab + nab-paclitaxel + ddAC	55	43.6 (24/55)	Study-specific pathological response; descriptive only
	Yam et al. (atezolizumab) [17]	Atezolizumab + nab-paclitaxel	33	Composite endpoint reported	Exploratory study-specific response; descriptive only
	NeoTENNIS [18]	Toripalimab + nab-paclitaxel followed by EC	70	55.7	Study-specific pCR; descriptive only
Platinum + immunotherapy regimens	NeoTRIPaPDL1 [15]	Atezolizumab + carboplatin + nab-paclitaxel	280 randomized (138 vs. 142)	48.6 vs. 44.4	Randomized regimen-level comparison; no isolated component effect inferred
	cTRIO [19]	Tislelizumab + nab-paclitaxel + carboplatin	62	56.5	Study-specific pCR; descriptive only
	CamRelief [20]	Camrelizumab + carboplatin + nab-paclitaxel	441 randomized (222 vs. 219)	56.8 vs. 44.7	Randomized regimen-level comparison
	NeoSAC [25]	Nab-paclitaxel-based intensified combination regimen	34	70.6	ypT0/Tis ypN0; descriptive only
Immunotherapy + targeted therapy + chemotherapy	NeoPanDa03 [21]	Camrelizumab + apatinib + nab-paclitaxel-based chemotherapy	34	67.6	Study-specific pCR; descriptive only
Platinum + immunotherapy regimens	Kuemmel et al. [26]	Tiragolumab + atezolizumab + nab-paclitaxel-containing therapy + carboplatin vs. same regimen without carboplatin	42 (22 vs. 20)	45.5 vs. 55.0	Separate exploratory randomized regimen-level comparison; not pooled with the four checkpoint-inhibitor trials
Immunotherapy-containing/mixed cohorts	KEYNOTE-173 [27]	Pembrolizumab + nab-paclitaxel; selected cohorts also included carboplatin	40 eligible	65.0 (26/40)	Study-level descriptive pCR across eligible nab-paclitaxel cohorts A–D; no comparative pooling
Immunotherapy-containing/mixed cohorts	NeoImmunoboost [28]	Pembrolizumab + nab-paclitaxel-containing chemotherapy	50	66.0 (33/50)	Prospective descriptive pCR
Immunotherapy-containing/mixed cohorts	Wang et al. [29]	Camrelizumab + nab-paclitaxel + epirubicin	39	64.1 (25/39)	Prospective descriptive pCR
Platinum + immunotherapy regimens	Chen et al. [30]	SBRT + adebrelimab followed by adebrelimab + nab-paclitaxel + carboplatin	13 recruited; 10 modified-ITT efficacy-evaluable	90.0 (9/10)	Prospective pilot; sensitivity exclusion because of SBRT/small n
Platinum-containing regimens	Huang et al. [31]	Nab-paclitaxel + carboplatin	TNBC subgroup n = 2	50.0 (1/2)	Separately extractable tiny TNBC subgroup; sensitivity exclusion
Immunotherapy-containing/mixed cohorts	Zheng et al. [32]	Camrelizumab + nab-paclitaxel-containing non-platinum chemotherapy	20 surgery/evaluable	65.0 (13/20)	Prospective descriptive pCR; denominator sensitivity
Immunotherapy-containing/mixed cohorts	TREND [33]	Tislelizumab + nab-paclitaxel followed by EC	44 efficacy-evaluable	68.2 (30/44)	Prospective descriptive pCR; denominator sensitivity

**Table 4:** Study-Level Data Used in the Quantitative Meta-Analyses.

Study	Design	Analysis	pCR Definition	Analytic Population	Events/Total Intervention or Single Arm	Events/Total Control	Data Source
IMpassion031 [13]	Randomized trial	Checkpoint-inhibitor RR	ypT0/is ypN0	ITT/all randomized	95/165	69/168	Primary report
GeparNuevo [14]	Randomized trial	Checkpoint-inhibitor RR	ypT0 ypN0	Randomized efficacy population	47/88	38/86	Primary report
NeoTRIPaPDL1 [15]	Randomized trial	Checkpoint-inhibitor RR	No residual invasive disease in breast and nodes	ITT	67/138	63/142	Primary report
CamRelief [20]	Randomized trial	Checkpoint-inhibitor RR	ypT0/Tis ypN0	ITT; no surgery imputed non-pCR	126/222	98/219	Primary report
Yuan et al. [8]	Prospective single-arm	Pooled proportion	ypT0/Tis N0	67 evaluable eligible patients	32/67	—	Primary report
Mrózek et al. (TNBC subgroup) [9]	Prospective single-arm	Pooled proportion	pCR in breast and axilla	TNBC subgroup	6/12	—	Primary report
NeoTENNIS [18]	Prospective single-arm	Pooled proportion	tpCR: ypT0/is ypN0	ITT; all 70 enrolled	39/70	—	Primary report
cTRIO [19]	Prospective single-arm	Pooled proportion	ypT0/Tis ypN0	All 62 enrolled	35/62	—	Primary report
NeoSAC [25]	Prospective single-arm	Pooled proportion	ypT0/Tis ypN0	ITT; all 34 enrolled	24/34	—	Primary report
NeoPanDa03 [21]	Prospective single-arm	Pooled proportion	tpCR: ypT0/is ypN0	34 pCR-evaluable patients	23/34	—	Primary report
KEYNOTE-173 A-D [27]	Prospective multicohort	Pooled proportion	ypT0/is ypN0	Eligible nab-paclitaxel cohorts A-D combined	26/40	—	Primary report/cohort linkage
Foldi et al. [16]	Prospective phase I/II	Pooled proportion	ypT0/is ypN0	Phase II ITT at recommended dose (n = 55)	24/55	—	Primary report
NeoImmunoboost [28]	Prospective phase II	Pooled proportion	ypT0/is ypN0	Enrolled/treated	33/50	—	Primary report
Wang et al. [29]	Prospective phase II	Pooled proportion	pCR	Enrolled/treated	25/39	—	Primary report
TREND [33]	Prospective phase II	Pooled proportion	pCR	Efficacy-evaluable	30/44	—	Primary report
Chen et al. [30]	Prospective pilot	Pooled proportion	ypT0/is ypN0	Modified-ITT efficacy-evaluable (10/13 recruited)	9/10	—	Primary report
Huang et al. [31]	Prospective phase II	Pooled proportion	ypT0/is ypN0	Separately reported TNBC subgroup	1/2	—	Primary report
Zheng et al. [32]	Prospective phase II	Pooled proportion	ypT0/is ypN0	Surgery/evaluable	13/20	—	Primary report

Note: WSG-ADAPT-TN was analyzed separately as a randomized comparison of nab-paclitaxel plus carboplatin versus nab-paclitaxel plus gemcitabine and was not included in the checkpoint-inhibitor pooled RR.

**Table 5:** Comparative Evidence by Clinically Coherent Randomized Treatment Contrast and Leave-One-Out Sensitivity Analysis.

Analysis	N	Effect	95% CI	I^2^ (%)	Note
Checkpoint inhibitor pooled RR [13,14,15,20]	4	RR = 1.25	1.12–1.41	0	ICI + matched chemotherapy vs. control
WSG-ADAPT-TN [7]	1	45.9% vs. 28.7%	—	—	Carboplatin vs. gemcitabine; not pooled
Prospective non-randomized pooled pCR [8,9,16,18,19,21,25,27,28,29,30,31,32,33]	14	pCR = 59.8%	53.8–65.6	32.7	REML + HKSJ; descriptive
Leave-one-out: IMpassion031 omitted	3	RR = 1.20	1.05–1.38	0.0	Sensitivity analysis
Leave-one-out: GeparNuevo omitted	3	RR = 1.26	1.11–1.43	4	Sensitivity analysis
Leave-one-out: NeoTRIPaPDL1 omitted	3	RR = 1.30	1.14–1.48	0.0	Sensitivity analysis
Leave-one-out: CamRelief omitted	3	RR = 1.24	1.07–1.45	5.9	Sensitivity analysis

Note: For the leave-one-out sensitivity analysis, each contributing randomized checkpoint-inhibitor trial was omitted sequentially. RRs were recalculated using the same DerSimonian–Laird random-effects model applied in the primary analysis. CI, confidence interval; HKSJ, Hartung-Knapp-Sidik-Jonkman; ICI, immune checkpoint inhibitor; I^2^, heterogeneity statistic; pCR, pathological complete response; REML, restricted maximum likelihood; RR, risk ratio.

**Table 6:** Summary of Quantitative Meta-Analysis Outcomes.

Analysis	Studies (n)	Total Patients	Effect Size	95% CI	I^2^ (%)	*p*-Value
Checkpoint inhibitor pooled RR for pCR [13,14,15,20]	4	1228	RR = 1.25	1.12–1.41	0	<0.001
Prospective non-randomized pooled pCR proportion [8,9,16,18,19,21,25,27,28,29,30,31,32,33]	14	539	59.8%	53.8–65.6	32.7	—

## Data Availability

The original contributions presented in this study are included in the article and Supplementary Materials. The study-level data used for the meta-analysis were extracted from the published studies included in this systematic review. The extracted study-level dataset underlying the meta-analytic analyses is available from the corresponding author upon reasonable request. Further inquiries can be directed to the corresponding author.

## Supplementary Materials

The following supporting information can be downloaded at: https://www.mdpi.com/article/10.3390/jcm15187321/s1, Supplementary File S1: Search Strategies, Platforms, Execution Dates, Search Yields, and Record Management; Supplementary Table S1: Final Manifest of the 21 Included Studies; Supplementary Table S2: Report-Level Exclusions after Full-Text/Report-Level Assessment; Supplementary File S2: PRISMA 2020 Checklist [22].

## Abbreviations

The following abbreviations are used in this manuscript:

AC	Doxorubicin/Cyclophosphamide
CENTRAL	Cochrane Central Register of Controlled Trials
CI	Confidence Interval
ddAC	Dose-Dense Doxorubicin/Cyclophosphamide
DNA	Deoxyribonucleic Acid
EC	Epirubicin/Cyclophosphamide
EFS	Event-Free Survival
ER	Estrogen Receptor
GRADE	Grading of Recommendations Assessment, Development and Evaluation
HER2	Human Epidermal Growth Factor Receptor 2
I^2^	Higgins Heterogeneity Statistic
ITT	Intention-to-Treat
JBI	Joanna Briggs Institute
MeSH	Medical Subject Headings
OS	Overall Survival
PD-1	Programmed Cell Death Protein 1
PD-L1	Programmed Death-Ligand 1
pCR	Pathological Complete Response
PR	Progesterone Receptor
PRISMA	Preferred Reporting Items for Systematic Reviews and Meta-Analyses
PROSPERO	International Prospective Register of Systematic Reviews
RCB	Residual Cancer Burden
RCT	Randomized Controlled Trial
REML	Restricted Maximum Likelihood
RoB 2	Cochrane Risk of Bias 2 Tool
RR	Risk Ratio
TNBC	Triple-Negative Breast Cancer
tpCR	Total Pathological Complete Response
VEGFR-2	Vascular Endothelial Growth Factor Receptor 2
ypN0	No Residual Pathological Cancer in Regional Lymph Nodes After Neoadjuvant Therapy
ypT0/is	No Residual Invasive Tumor in the Breast (With or Without Residual In Situ Disease) After Neoadjuvant Therapy
