# Supplementary material for: Pathological Complete Response to Nab-Paclitaxel–Containing Neoadjuvant Regimens in Early Triple-Negative Breast Cancer: A Systematic Review and Meta-Analysis
Source: J Clin Med. 2026 Sep 21;15(18):7321. doi: 10.3390/jcm15187321 (PMC13608542; doi:10.3390/jcm15187321)
Supplement: Supplementary file 1 [file jcm-15-07321-s001.zip › jcm-4453464-supplementary.pdf]

Supplementary File S1

Search Strategies, Platforms, Execution Dates, Search Yields, and Record Management

This supplement documents the final refined rerun used for the revised systematic review. The eligibility criteria and publication cutoff (31 March 2026) were unchanged. The electronic retrieval was refined for sensitivity by avoiding a mandatory pCR concept in the final search logic where documented and by using broader nab-paclitaxel/platinum/checkpoint-inhibitor terminology. The final five-source retrieval comprised 1,224 records.

S1.1 PubMed/MEDLINE

Platform/provider: NCBI PubMed web interface

Execution date: 8 September 2026

Final raw retrieval: n = 103

Records published after 31 March 2026 removed before screening: n = 2

Documented executed reconciliation query:

("triple negative breast cancer" OR TNBC) AND (neoadjuvant OR preoperative) AND ("nab-paclitaxel" OR "albumin-bound paclitaxel") AND (platinum OR carboplatin OR cisplatin OR immunotherapy OR pembrolizumab OR atezolizumab OR durvalumab OR camrelizumab OR toripalimab OR tislelizumab OR "PD-1" OR "PD-L1")

The stated publication cutoff was retained during record management. The archived PubMed CSV contains 103 records and is the record-level audit source.

S1.2 Scopus

Platform/provider: Elsevier Scopus

Execution date: 9 September 2026

Final raw retrieval: n = 117

Records published after 31 March 2026 removed before screening: n = 6

Executed query:

TITLE-ABS-KEY("triple negative breast cancer" OR "triple-negative breast cancer" OR TNBC) AND TITLE-ABS-KEY(neoadjuvant OR "neo-adjuvant" OR preoperative) AND TITLE-ABS-KEY("nab-paclitaxel" OR "nab paclitaxel" OR nabpaclitaxel OR "albumin-bound paclitaxel" OR "albumin bound paclitaxel" OR "nanoparticle albumin-bound paclitaxel" OR Abraxane) AND TITLE-ABS-KEY(carboplatin OR cisplatin OR platinum OR pembrolizumab OR atezolizumab OR durvalumab OR camrelizumab OR tislelizumab OR nivolumab OR "immune checkpoint inhibitor" OR "immune checkpoint inhibitors" OR "checkpoint inhibitor" OR "checkpoint inhibitors" OR immunotherap*)

Limits: publication years 2005–2026; the exact 31 March 2026 cutoff was enforced during record management.

S1.3 CENTRAL

Platform/provider: Cochrane Library, Search Manager; database: CENTRAL

Execution date: 8 September 2026

Limits: Trials; Publication Year 2005–2026; Cochrane Library publication date January 2005–March 2026

Final retrieval: n = 140 trial records

#1 ("triple negative breast cancer" OR "triple-negative breast cancer" OR TNBC):ti,ab,kw

#2 (neoadjuvant OR "neo-adjuvant" OR preoperative):ti,ab,kw

#3 ("nab-paclitaxel" OR "nab paclitaxel" OR nabpaclitaxel OR "albumin-bound paclitaxel" OR "albumin bound paclitaxel" OR "nanoparticle albumin-bound paclitaxel" OR Abraxane):ti,ab,kw

#7 (carboplatin OR cisplatin OR platinum OR pembrolizumab OR atezolizumab OR durvalumab OR camrelizumab OR tislelizumab OR nivolumab OR "immune checkpoint inhibitor" OR "immune checkpoint inhibitors" OR "checkpoint inhibitor" OR "checkpoint inhibitors" OR immunotherap*):ti,ab,kw

#8 #1 AND #2 AND #3 AND #7

No pCR term was required in the finalized CENTRAL electronic query; pCR eligibility was adjudicated during screening/full-text assessment.

S1.4 Embase

Platform/provider: Embase.com (Elsevier)

Execution date of documented rerun: 10 September 2026

Final raw retrieval: n = 564 records

Records published after the eligibility cutoff of 31 March 2026 removed before screening: n = 19

Date-eligible Embase records entering deduplication: n = 545

Executed rerun strategy:

('triple negative breast cancer'/exp OR 'triple negative breast cancer':ti,ab,kw OR 'triple-negative breast cancer':ti,ab,kw OR TNBC:ti,ab,kw) AND ('nab paclitaxel'/exp OR 'nab-paclitaxel':ti,ab,kw OR 'nab paclitaxel':ti,ab,kw OR nabpaclitaxel:ti,ab,kw OR 'albumin-bound paclitaxel':ti,ab,kw OR 'albumin bound paclitaxel':ti,ab,kw OR 'nanoparticle albumin-bound paclitaxel':ti,ab,kw OR 'nanoparticle albumin bound paclitaxel':ti,ab,kw OR Abraxane:ti,ab,kw) AND (neoadjuvant:ti,ab,kw OR 'neo-adjuvant':ti,ab,kw OR preoperative:ti,ab,kw) AND (platinum:ti,ab,kw OR carboplatin:ti,ab,kw OR cisplatin:ti,ab,kw OR pembrolizumab:ti,ab,kw OR atezolizumab:ti,ab,kw OR durvalumab:ti,ab,kw OR camrelizumab:ti,ab,kw OR tislelizumab:ti,ab,kw OR nivolumab:ti,ab,kw OR 'immune checkpoint inhibitor':ti,ab,kw OR 'immune checkpoint inhibitors':ti,ab,kw OR 'checkpoint inhibitor':ti,ab,kw OR 'checkpoint inhibitors':ti,ab,kw OR immunotherap*:ti,ab,kw)

No pCR/pathological complete response term was required in the rerun electronic query; pCR eligibility was adjudicated during screening/full-text assessment. The publication eligibility window remained January 2005 through 31 March 2026. Records indexed in 2026 but published after 31 March 2026 were removed during record management rather than being used to alter the review cutoff.

Audit note: The rerun returned n = 564 records, matching the archived Embase export used in the revised review. The rerun export is retained as the record-level audit source. The search strategy, platform, execution date, yield, and cutoff handling are therefore documented for reproducibility.

S1.5 Google Scholar

Platform/provider: Google Scholar

Execution date: 9 September 2026

Query: "triple negative breast cancer" "nab-paclitaxel" neoadjuvant

Procedure: custom year range 2005–2026; default relevance ranking; patents and citations excluded.

Records captured and screened: n = 300 (30 relevance-ranked result pages; 10 results per page)

Records published after 31 March 2026 removed before screening: n = 10

Google Scholar was screened to a depth of 300 relevance-ranked results (30 pages, 10 results per page). The screening denominator therefore uses n = 300.

S1.6 Final source yields and cutoff handling

Source	Raw records	Post-cutoff removed	Date-eligible records
PubMed/MEDLINE	103	2	101
Scopus	117	6	111
CENTRAL	140	0	140
Embase	564	19	545
Google Scholar	300	10	290
Total	1,224	37	1,187

S1.7 Record management and deduplication

The 37 records published after the 31 March 2026 cutoff were removed before deduplication. The remaining 1,187 date-eligible source records were deduplicated at report level. Normalized DOI was prioritized where available, followed by normalized exact-title matching; publication year/author information and manual adjudication were used for ambiguous same-report matches. Distinct publications from the same trial were retained as separate reports and subsequently linked at the study-family stage. A total of 326 duplicate source records were removed, leaving 861 unique records for title/abstract screening.

S1.8 Screening outcome

Of 861 unique records screened, 748 were excluded at title/abstract level. A total of 113 reports were sought for retrieval; none was not retrieved. All 113 reports were assessed for eligibility. Ninety-two reports were excluded with documented report-level reasons, and 21 unique studies represented by 21 primary reports were included. The complete individual report-level exclusion ledger is provided in Supplementary Table S2.

S1.9 Audit statement

All five sources are now documented with source/platform information, execution information, search strategy or capture procedure, raw yield, and cutoff handling. The Embase rerun returned n = 564, matching the archived export, and the rerun strategy is reported verbatim in Section S1.4.

S1.10 Full-text exclusion summary

For concise reporting in the PRISMA flow diagram, the 92 full-text exclusions were grouped into five mutually exclusive categories. The complete report-level audit, including the specific exclusion reason for each of the 92 reports, is provided in Supplementary Table S2.

Clubbed exclusion category	Reports, n
Secondary/companion/duplicate report of an included study	45
Conference/meeting abstract only	23
Trial registration/protocol/no eligible results	13
Ineligible intervention/outcome for this review	8
Ineligible design/ancillary non-efficacy report	3
Total full-text exclusions	92

The grouped categories above are used only for concise PRISMA presentation; the operative report-level exclusion decisions are listed in Supplementary Table S2.

S1.11 Audit statement

All five sources are documented with source/platform information, execution information, search strategy or capture procedure, raw yield, cutoff handling, record management and deduplication. The PRISMA screening count is 861 unique records, 748 title/abstract exclusions, 113 reports assessed, 92 full-text exclusions, and 21 included studies. The 92 full-text exclusions are grouped for PRISMA readability while the complete individual report-level audit is provided in Supplementary Table S2.

Supplementary Table S1. Final manifest of the 21 included studies

This table is the final study-family manifest used for the regenerated PRISMA flow, study-characteristics table, and quantitative analyses. It records the primary report, trial identifier where available, eligible regimen, analysis denominator, pCR event count, study-specific pCR definition, and final synthesis role. Companion reports are linked to the study family and are not counted as independent studies.

Final synthesis architecture

Synthesis component	Studies, n	Study families
Prospective non-randomized descriptive pCR meta-analysis	14	Yuan; Mrózek; NeoTENNIS; cTRIO; NeoSAC; NeoPanDa03; KEYNOTE-173; Foldi/durvalumab; NeoImmunoboost/AGO-B-041; Wang/NCT04213898; TREND; Chen/NCT05132790; Huang 2015; Zheng/NCT04676997
Checkpoint-inhibitor randomized RR meta-analysis	4	IMpassion031; GeparNuevo; NeoTRIPaPDL1; CamRelief
Randomized evidence reported separately	2	WSG-ADAPT-TN; Kuemmel/CO42177
Qualitative-only eligible study	1	Yam/atezolizumab; exact integer pCR numerator not reconstructed from a rounded percentage

No.	Study / trial	Year / design	Primary report / DOI	Trial identifier	Eligible neoadjuvant regimen	Analysis denominator	pCR result	Study-specific pCR definition	Role in final synthesis
1	WSG-ADAPT-TN	2018; Randomized phase II	Comparison of neoadjuvant nab-paclitaxel+ carboplatin vs nab-paclitaxel+ gemcitabine in triple-negative breast cancer: randomized WSG-ADAPT-TN trial results DOI: 10.1093/jnci/djx258	NCT01815242	nab-paclitaxel + carboplatin vs nab-paclitaxel + gemcitabine	154 vs 182	45.9% vs 28.7%	ypT0/is ypN0	Randomized separate evidence
2	Yuan	2021; Prospective phase II	Phase II Trial of Neoadjuvant Carboplatin and Nab-Paclitaxel in Patients with Triple-Negative Breast Cancer DOI: 10.1002/onco.13574	Not reported in final manifest	nab-paclitaxel + carboplatin-containing neoadjuvant regimen	67	32/67	ypT0/Tis N0	Nonrandomized proportion
3	Mrózek	2014; Prospective study; mixed breast-cancer population	Phase II trial of neoadjuvant weekly nanoparticle albumin-bound paclitaxel, carboplatin, and biweekly bevacizumab therapy in women with clinical stage II or III HER2-negative breast cancer DOI: 10.1016/j.clbc.2014.02.005	Not reported in final manifest	nab-paclitaxel-containing regimen; TNBC subgroup extractable	12 TNBC	6/12	pCR in breast and axilla; 6 pCRs, all in TNBC	Nonrandomized proportion
4	IMpassion031	2020; Randomized phase III	Neoadjuvant atezolizumab in combination with sequential nab-paclitaxel and anthracycline-based chemotherapy versus placebo and chemotherapy in patients with early-stage triple-negative breast cancer (IMpassion031): a randomised, double-blind, phase 3 trial DOI: 10.1016/s0140-6736(20)31953-x	Not reported in final manifest	atezolizumab + nab-paclitaxel→AC vs placebo + same chemotherapy	165 vs 168	95/165 vs 69/168	ypT0/is ypN0	4-RCT RR

No.	Study / trial	Year / design	Primary report / DOI	Trial identifier	Eligible neoadjuvant regimen	Analysis denominator	pCR result	Study-specific pCR definition	Role in final synthesis
5	GeparNuevo	2019; Randomized phase II	A randomised phase II study investigating durvalumab in addition to an anthracycline taxane-based neoadjuvant therapy in early triple-negative breast cancer: clinical results and biomarker analysis of GeparNuevo study<br>DOI: 10.1093/annonc/mdz158	NCT02685059	durvalumab + nab-paclitaxel→EC vs placebo + same chemotherapy	88 vs 86	47/88 vs 38/86	ypT0 ypN0	4-RCT RR
6	Foldi / durvalumab	2021; Prospective single-arm	Neoadjuvant durvalumab plus weekly nab-paclitaxel and dose-dense doxorubicin/cyclophosphamide in triple-negative breast cancer<br>DOI: 10.1038/s41523-021-00219-7	NCT02489448	durvalumab + weekly nab-paclitaxel→ddAC	55	24/55	ypT0/is ypN0	Nonrandomized proportion
7	Yam / atezolizumab	2023; Prospective single-arm	A phase II study of neoadjuvant atezolizumab and nab-paclitaxel in patients with anthracycline-resistant early-stage triple-negative breast cancer<br>DOI: 10.1007/s10549-023-06929-9	Not reported in final manifest	atezolizumab + nab-paclitaxel-containing neoadjuvant regimen	37	~30% reported	Pure pCR numerator not directly extractable from rounded rate	Qualitative only
8	NeoTENNIS	2024; Prospective phase II	Neoadjuvant anthracycline followed by toripalimab combined with nab-paclitaxel in patients with early triple-negative breast cancer (NeoTENNIS): a single-arm, phase II study<br>DOI: 10.1016/j.eclinm.2024.102700	NCT04418154	eligible nab-paclitaxel-containing neoadjuvant regimen	70 ITT	39/70	tpCR = ypT0/is ypN0	Nonrandomized proportion
9	NeoTRIPaPDL1	2022; Randomized phase III	Pathologic complete response (pCR) to neoadjuvant treatment with or without atezolizumab in triple-negative, early high-risk and locally advanced breast cancer: NeoTRIP Michelangelo randomized study<br>DOI: 10.1016/j.annonc.2022.02.004	NCT02620280	atezolizumab + carboplatin/nab-paclitaxel vs chemotherapy	138 vs 142	67/138 vs 63/142	Primary pCR endpoint reported for breast and nodes; use published study-specific definition without harmonizing labels	4-RCT RR
10	cTRIO	2025; 2026; Prospective phase II	Neoadjuvant tislelizumab plus nab-paclitaxel and carboplatin for triple-negative breast cancer: a multicenter, open-label, phase II cTRIO study<br>DOI: 10.1016/j.eclinm.2025.103709	ChiCTR2100041675	tislelizumab + nab-paclitaxel + carboplatin	62 efficacy/ITT analysis set	35/62	ypT0/Tis ypN0	Nonrandomized proportion
11	CamRelief	2025; Randomized clinical trial	Camrelizumab vs Placebo in Combination With Chemotherapy as Neoadjuvant Treatment in Patients With Early or Locally Advanced Triple-Negative Breast Cancer: The CamRelief Randomized Clinical Trial<br>DOI: 10.1001/jama.2024.23560	NCT04613674	camrelizumab + chemotherapy vs placebo + chemotherapy; nab-paclitaxel-containing backbone	222 vs 219	126/222 vs 98/219	ypT0/Tis ypN0	4-RCT RR
12	NeoSAC	2025; Prospective study	Neoadjuvant apatinib addition to sintilimab and carboplatin-taxane based chemotherapy in patients with early triple-negative breast cancer: the phase 2 NeoSAC trial<br>DOI: 10.1038/s41392-025-02137-7	Not reported in final manifest	eligible nab-paclitaxel-containing neoadjuvant regimen	34 ITT (33 pathologically evaluable; one lost to follow-up)	24/34	ypT0/Tis ypN0	Nonrandomized proportion

No.	Study / trial	Year / design	Primary report / DOI	Trial identifier	Eligible neoadjuvant regimen	Analysis denominator	pCR result	Study-specific pCR definition	Role in final synthesis
13	NeoPanDa03	2025; Prospective study	Exploratory phase II trial of an anti-PD-1 antibody camrelizumab combined with a VEGFR-2 inhibitor apatinib and chemotherapy as a neoadjuvant therapy for triple-negative breast cancer (NeoPanDa03): efficacy, safety and biomarker analysis DOI: 10.1038/s41392-025-02337-1	NCT05447702	eligible nab-paclitaxel-containing neoadjuvant regimen	34 efficacy-evaluable	23/34	tpCR = ypT0/is ypN0	Nonrandomized proportion
14	Kuemmel	2025; Randomized/exploratory early-stage cohort within multicohort report	Safety and efficacy of tiragolumab, atezolizumab and chemotherapy for early-stage or PD-L1-positive advanced triple-negative breast cancer: a phase Ib study DOI: 10.1016/j.esmoop.2025.105869	NCT04584112	nab-paclitaxel-containing immunotherapy strategy with platinum/no-platinum contrast	42 randomized: 22 platinum vs 20 non-platinum	10/22 vs 11/20	ypT0/is ypN0	Randomized separate evidence
15	KEYNOTE-173	2020; Phase Ib open-label multicohort	Pembrolizumab plus chemotherapy as neoadjuvant treatment of high-risk, early-stage triple-negative breast cancer: results from the phase 1b open-label, multicohort KEYNOTE-173 study DOI: 10.1016/j.annonc.2020.01.072	NCT02622074	pembrolizumab + chemotherapy; eligible nab-paclitaxel cohorts A–D	40 nab-paclitaxel cohorts A-D	26/40	ypT0/Tis ypN0	Nonrandomized proportion
16	NeoImmunoboost / AGO-B-041	2023; Single-arm phase II	Pembrolizumab in combination with nab-paclitaxel for the treatment of patients with early-stage triple-negative breast cancer - A single-arm phase II trial (NeoImmunoboost, AGO-B-041) DOI: 10.1016/j.ejca.2023.01.001	NCT03289819 / AGO-B-041	pembrolizumab + nab-paclitaxel-containing neoadjuvant chemotherapy	50	33/50	ypT0/is ypN0	Nonrandomized proportion
17	Wang / NCT04213898	2023; Prospective phase II	Neoadjuvant camrelizumab plus nab-paclitaxel and epirubicin in early triple-negative breast cancer: a single-arm phase II trial DOI: 10.1038/s41467-023-42479-w	NCT04213898	camrelizumab + nab-paclitaxel + epirubicin	39	25/39	ypT0/is ypN0	Nonrandomized proportion
18	TREND	2025; Prospective single-arm phase II	Efficacy, safety and exploratory analysis of neoadjuvant tislelizumab (a PD-1 inhibitor) plus nab-paclitaxel followed by epirubicin/cyclophosphamide for triple-negative breast cancer: a phase 2 TREND trial DOI: 10.1038/s41392-025-02254-3	ChiCTR2000035262	tislelizumab + nab-paclitaxel→EC	53 enrolled; 44 efficacy-evaluable	30/44	ypT0/Tis ypN0	Nonrandomized proportion
19	Chen / NCT05132790	2023; Prospective single-arm pilot	Effects of neoadjuvant stereotactic body radiotherapy plus adebrelimab and chemotherapy for triple-negative breast cancer: A pilot study DOI: 10.7554/elife.91737	NCT05132790	adebrelimab + SBRT then adebrelimab + nab-paclitaxel + carboplatin	13 treated; 10 modified-ITT efficacy-evaluable	9/10	ypT0/is ypN0	Nonrandomized proportion
20	Huang 2015	2015; Prospective phase II; mixed breast-cancer population	Phase II trial of weekly nab-paclitaxel and carboplatin treatment with or without trastuzumab as nonanthracycline neoadjuvant chemotherapy for locally advanced breast cancer DOI: 10.2147/ijn.s77000	Not reported in final manifest	nab-paclitaxel-containing regimen; separately extractable TNBC subgroup	2 TNBC patients in the nab-paclitaxel cohort	1/2	ypT0/is ypN0	Nonrandomized proportion

No.	Study / trial	Year / design	Primary report / DOI	Trial identifier	Eligible neoadjuvant regimen	Analysis denominator	pCR result	Study-specific pCR definition	Role in final synthesis
21	Zheng / NCT04676997	2024; Prospective phase II	Clinical efficacy and biomarker analysis of neoadjuvant camrelizumab plus chemotherapy for early-stage triple-negative breast cancer: a experimental single-arm phase II clinical trial pilot study DOI: 10.1097/js9.0000000000001011	NCT04676997	camrelizumab + non-platinum chemotherapy containing nab-paclitaxel	23 treated; 20 surgery/evaluable	13/20	ypT0/is ypN0	Nonrandomized proportion

Notes: pCR definitions are preserved as reported by each study. Chen/NCT05132790 contributed 9/10 pCR among the modified-ITT efficacy-evaluable population after SBRT plus adebrelimab and nab-paclitaxel/carboplatin. Huang 2015 contributed a separately extractable TNBC subgroup of n=2 (1/2 pCR) and is flagged for tiny-subgroup sensitivity analysis. NeoSAC is presented as 24/34 in the ITT population (one patient was lost to follow-up before pathological assessment). Yam/atezolizumab is qualitative only because an exact pure-pCR numerator was not directly extractable.

Abbreviations: AC, doxorubicin/cyclophosphamide; ICI, immune checkpoint inhibitor; ITT, intention-to-treat; pCR, pathological complete response; RR, risk ratio; SBRT, stereotactic body radiotherapy; TNBC, triple-negative breast cancer.

Study-level quantitative eligibility and denominator ledger for prospective non-randomized evidence

Study / trial	Enrolled / relevant N	Excluded / non-evaluable	Analytic N used for pCR	pCR events	pCR definition	Quantitative disposition / reason
Yuan	67 eligible evaluable	None reported for relevant cohort	67	32	ypT0/Tis N0	Included; eligible prospective TNBC cohort with pure pCR numerator/denominator.
Mrózek TNBC subgroup	33 enrolled overall; 12 TNBC	Non-TNBC participants outside relevant subgroup	12 TNBC	6	pCR in breast and axilla	Included; separately extractable TNBC subgroup.
Foldi / durvalumab	55 in phase II ITT population at recommended dose	None material for reported primary pCR denominator	55	24	ypT0/is ypN0	Included; eligible prospective single-arm dataset.
Yam / atezolizumab	37 reported study population	Pure integer pCR numerator not directly extractable from rounded/composite reporting	Not used quantitatively	—	Composite/pathological response reporting	Qualitative only; pure pCR numerator not directly extractable without back-calculation.
NeoTENNIS	70 enrolled	None; ITT denominator retained	70	39	tpCR = ypT0/is ypN0	Included; ITT/all-enrolled denominator.
cTRIO	62 analysis set	None material for reported analysis set	62	35	ypT0/Tis ypN0	Included; reported analysis denominator retained.
NeoSAC	34 enrolled/ITT	1 lost to follow-up, but retained in ITT denominator	34	24	ypT0/Tis ypN0	Included; published ITT pCR denominator retained.
NeoPanDa03	35 enrolled	1 withdrew because of treatment intolerance and was excluded from efficacy denominator	34	23	tpCR = ypT0/is ypN0	Included using reported efficacy denominator; denominator-selection limitation acknowledged.
KEYNOTE-173 cohorts A–D	40 eligible nab-paclitaxel cohorts	Cohorts E–F excluded because they used solvent-based paclitaxel	40	26	ypT0/is ypN0	Included; eligible nab-paclitaxel cohorts A–D combined to avoid within-study double counting.

Study / trial	Enrolled / relevant N	Excluded / non-evaluable	Analytic N used for pCR	pCR events	pCR definition	Quantitative disposition / reason
NeoImmunoboost / AGO-B-041	50 treated	None reported for relevant primary pCR denominator	50	33	ypT0/is ypN0	Included; eligible prospective single-arm dataset.
Wang / NCT04213898	39 treated	None reported for relevant primary pCR denominator	39	25	pCR	Included; eligible prospective single-arm dataset.
TREND	53 enrolled	9 not included in reported efficacy-evaluable population	44	30	ypT0/Tis ypN0	Included using study-reported efficacy denominator; tested in denominator sensitivity analysis.
Chen / NCT05132790	13 treated	3 not included in modified-ITT efficacy population	10	9	ypT0/is ypN0	Included using study-reported modified-ITT denominator; tested in denominator sensitivity analysis.
Huang 2015 TNBC subgroup	30 in overall nab-paclitaxel cohort; 2 TNBC	Non-TNBC participants outside relevant subgroup	2 TNBC	1	ypT0/is ypN0	Included; separately extractable TNBC subgroup; tiny-subgroup sensitivity analysis performed.
Zheng / NCT04676997	23 treated	3 not included in surgery/evaluable pCR population	20	13	ypT0/is ypN0	Included using surgery/evaluable denominator; tested in denominator sensitivity analysis.

Denominator policy: the denominator specified by the primary report for its pCR analysis was retained. Participants excluded by the primary report were not reclassified as non-pCR unless the report itself counted them as nonresponders. Studies using efficacy- or surgery-evaluable denominators were examined in a dedicated sensitivity analysis. Separately extractable TNBC subgroups were eligible when numerator and denominator were both reported.

Supplementary Table S2. Report-level exclusions after full-text/report-level assessment

This table provides the final audited report-level exclusion ledger for the refined search. Ninety-two reports were excluded after detailed assessment. Each row preserves the report identity and the specific documented reason for exclusion. Distinct reports from the same underlying trial were retained at the report level and linked to the study family where applicable. The four studies specifically identified by Reviewer 1 (NeoImmunoboost/AGO-B-041, Wang/NCT04213898, Zheng/NCT04676997, and TREND) were adjudicated as eligible and therefore do not appear in this exclusion table.

Summary of report-level exclusion categories

Consolidated category	Reports, n
Secondary/companion/duplicate report of an included study	45
Conference/meeting abstract only	23
Trial registration/protocol/no eligible results	13
Ineligible intervention/no eligible nab-paclitaxel	4
No platinum or immune checkpoint inhibitor	2
Nab-paclitaxel-specific pCR not separately extractable	2
Retrospective/real-world design	2
Ancillary intervention/non-efficacy report	1

No.	Year	Report/citation identification	DOI / trial or source identifier	Source(s)	Study family	Specific reason for exclusion
1	2023	Nab-paclitaxel Followed by Dose-dense Epirubicin/Cyclophosphamide in Neoadjuvant Chemotherapy for Triple-negative Breast Cancer: A Phase II Study	DOI: 10.1093/oncolo/oyac223; PMID:36426808; GS:16	Embase; Google Scholar; PubMed	—	No platinum or immune checkpoint inhibitor in the treatment strategy
2	2022	Subgroup analysis of Japanese patients in a phase III randomized, controlled study of neoadjuvant atezolizumab or placebo, combined with nab-paclitaxel and anthracycline-based chemotherapy in early triple-negative breast cancer (IMpassion031)	DOI: 10.1093/jjco/hyac098; PMID:35750038; PMID:35750038; CENTRAL:CN-02413698	CENTRAL; Embase; PubMed; Scopus	IMpassion031	Secondary/companion report of an included study
3	2022	Predictive Markers of Response to Neoadjuvant Durvalumab with Nab-Paclitaxel and Dose-Dense Doxorubicin/Cyclophosphamide in Basal-Like Triple-Negative Breast Cancer	DOI: 10.1158/1078-0432.ccr-21-3215; PMID:35377948; PMID:35377948; CENTRAL:CN-02399766	CENTRAL; Embase; PubMed; Scopus	—	Secondary/companion report of an included study
4	2022	Author Correction: Neoadjuvant durvalumab plus weekly nab-paclitaxel and dose-dense doxorubicin/cyclophosphamide in triple-negative breast cancer (npj Breast Cancer, (2021), 7, 1, (9), 10.1038/s41523-021-00219-7)	DOI: 10.1038/s41523-022-00392-3; PMID:35115541; EID:2-s2.0-85124396640; GS:252	Embase; Google Scholar; PubMed; Scopus	Foldi / durvalumab	Secondary/companion report of an included study
5	2026	A Single-Arm, Single-Center, Phase II Clinical Study of Camrelizumab Combined With Radiochemotherapy as Neoadjuvant Therapy for Early-Stage Triple-Negative Breast Cancer	Not available in audit record	Embase	—	Trial registration/protocol; no results available
6	2025	The preliminary efficacy and safety results of neoadjuvant phase II study of anlotinib plus tislelizumab combined with chemotherapy in triple-negative breast cancer.	DOI: 10.1200/jco.2025.43.16_suppl.e12600; GS:275	Embase; Google Scholar	—	Conference/meeting abstract only; no eligible full-text primary article
7	2025	Neoadjuvant penpulimab combined with taxanes and carboplatin in triple-negative breast cancer: A single-arm, open-label, multi-center phase II clinical study (neoTAPPL).	DOI: 10.1200/jco.2025.43.16_suppl.588; GS:255	Embase; Google Scholar	—	Mixed taxane regimen; nab-paclitaxel-specific pCR not separately extractable
8	2025	Neoadjuvant chemotherapy plus camrelizumab vs. chemotherapy in patients with locally advanced immunomodulatory triple-negative breast cancer: a prospective, randomized, open-label, phase 2 trial	DOI: 10.1158/1557-3265.sabcs24-p2-11-06	Embase	—	Conference/meeting abstract only; no eligible full-text primary article
9	2025	Real world experience with Carboplatin plus Nab-paclitaxel as neoadjuvant therapy in patients with early triple negative breast cancer	DOI: 10.1158/1557-3265.sabcs24-p5-09-16	Embase	—	Retrospective/real-world study; prospective design criterion not met
10	2025	A phase 2 trial combining tiragolumab and atezolizumab with neoadjuvant or first line chemotherapy for triple negative breast cancer (SKYLINE)	DOI: 10.1158/1557-3265.sabcs24-p2-12-23	Embase	—	Conference/meeting abstract only; no eligible full-text primary article
11	2024	A Single-Arm Phase II Trial of Neoadjuvant Toripalimab, Docetaxel, Plus Carboplatin in Patients With Localized Triple-Negative Breast Cancer	Not available in audit record	Embase	—	Wrong taxane/intervention; no nab-paclitaxel
12	2024	Efficacy, safety and exploratory analysis of neoadjuvant tislelizumab plus nab-paclitaxel followed by epirubicin/cyclophosphamide for TNBC: A phase II TREND trial	DOI: 10.1016/j.annonc.2024.08.271	Embase	TREND	Secondary/companion report of an included study
13	2024	The preliminary efficacy and safety results of neoadjuvant phase 2 study of penpulimab combined with taxanes and carboplatin in triple-negative breast cancer (neoTAPPL)	Not available in audit record	Embase	—	Mixed taxane regimen; nab-paclitaxel-specific pCR not separately extractable
14	2024	Neoadjuvant anthracycline followed by Toripalimab combined with nab-paclitaxel in patients with stage IIAIIIC triple negative breast cancer (NeoTENNIS): efficacy and safety results of a phase II study	DOI: 10.1158/1538-7445.sabcs23-po2-03-05	Embase	NeoTENNIS	Secondary/companion report of an included study

No.	Year	Report/citation identification	DOI / trial or source identifier	Source(s)	Study family	Specific reason for exclusion
15	2024	Camrelizumab in Combination with Nab-Paclitaxel and Carboplatin versus Nab-Paclitaxel and Carboplatin as Neoadjuvant Therapy for Triple Negative Breast Cancer: A Multicenter, Open-Label, Randomized Controlled Study	DOI: 10.1158/1538-7445.sabcs23-po1-03-11; CENTRAL:CN-02703337	CENTRAL; Embase	—	Conference/meeting abstract only; no eligible full-text primary article
16	2023	A Multicenter, Randomized Trial of Stereotactic Radiotherapy Combined With Adebrelimab and TCb (Nab-paclitaxel + Carboplatin) Versus Adebrelimab Combined With TCb (Nab-paclitaxel + Carboplatin) in Neoadjuvant Treatment of Triple-negative Breast Cancer	Not available in audit record	Embase	Chen / NCT05132790	Secondary/companion report of an included study
17	2023	Neoadjuvant tislelizumab plus nab-paclitaxel and carboplatin followed by adjuvant tislelizumab in patients with early triple-negative breast cancer.	DOI: 10.1200/jco.2023.41.16_suppl.602; GS:24	Embase; Google Scholar	—	Conference/meeting abstract only; no eligible full-text primary article
18	2023	Neoadjuvant tislelizumab, a PD-1 mAb plus nab-paclitaxel (nab-P) followed by tislelizumab plus epirubicin/cyclophosphamide (EC) for triple-negative breast cancers (TNBC): Interim analysis of clinical response in a phase 2 TREND trial	DOI: 10.1200/jco.2023.41.16_suppl.e14623; CENTRAL:CN-02773588	CENTRAL; Embase	TREND	Secondary/companion report of an included study
19	2023	Nab-paclitaxel followed by dose-dense epirubicin/cyclophosphamide in neoadjuvant chemotherapy for triple-negative breast cancer: an open-label, single-arm phase ii study	DOI: 10.1158/1538-7445.sabcs22-ot2-18-01	Embase	—	Conference/meeting abstract only; no eligible full-text primary article
20	2022	Phase II study of camrelizumab plus chemotherapy as neoadjuvant therapy in patients with early triple-negative breast cancer	DOI: 10.1016/j.annonc.2022.07.205	Embase	—	Conference/meeting abstract only; companion report of Wang/NCT04213898
21	2022	To Evaluate the Efficacy and Safety of Toripalimab Combined With Chemotherapy (Epirubicin + Cyclophosphamide → Nab-paclitaxel + Carboplatin) in the Neoadjuvant Treatment of Triple-negative Breast Cancer After High-intensity Focused Ultrasound (HIFU) Induction	Not available in audit record	Embase	—	Trial registration/protocol or no eligible full-text results report identified
22	2022	MO18-2 A phase II trial of neoadjuvant apatinib combined with nab-paclitaxel and carboplatin in triple-negative breast cancer	DOI: 10.1016/j.annonc.2022.05.141; GS:179	Embase; Google Scholar	—	Conference/meeting abstract only; no eligible full-text primary article
23	2022	A Single-arm, Prospective Phase II Study of Camrelizumab Plus Apatinib and Chemotherapy as Neoadjuvant Therapy for Triple Negative Breast Cancer (TNBC)	Not available in audit record	Embase	—	Trial registration/protocol; no eligible results report identified
24	2022	Anlotinib plus tislelizumab combined with chemotherapy as neoadjuvant treatment in triple-negative breast cancer: A prospective, single-arm, open-label phase II study	DOI: 10.1200/jco.2022.40.16_suppl.tps618	Embase	—	Conference/meeting abstract only; no eligible full-text primary article
25	2021	Neoadjuvant camrelizumab plus nab-paclitaxel and epirubicin for early triple-negative breast cancer: A single-arm, open-label, phase II study	DOI: 10.1016/j.annonc.2021.08.460	Embase	Wang / NCT04213898	Secondary/companion report of an included study
26	2021	A Multicenter, Open, Randomized Controlled Study of Camrelizumab+ Nab-paclitaxel + Carboplatin Versus Nab-paclitaxel + Carboplatin as Neoadjuvant Therapy for Triple Negative Breast Cancer (TNBC)	Not available in audit record	Embase	—	Trial registration/protocol or no eligible full-text results report identified
27	2021	PD-1 Inhibitor Sintilimab Concurrent With Epirubicin Cyclophosphamide and Nab-paclitaxel as Neoadjuvant Therapy for Triple Negative Breast Cancer	Not available in audit record	Embase	—	Trial registration/protocol; no eligible results report identified
28	2021	Neoadjuvant atezolizumab (atezo) and nab-paclitaxel (nab-p) in patients (pts) with triple-negative breast cancer (TNBC) with suboptimal clinical response to doxorubicin and cyclophosphamide (AC)	DOI: 10.1200/jco.2021.39.15_suppl.592; CENTRAL:CN-02305818	CENTRAL; Embase	—	Conference/meeting abstract only; no eligible full-text primary article
29	2020	3MO IMpassion031: Results from a phase III study of neoadjuvant (neoadj) atezolizumab + chemo in early triple-negative breast cancer (TNBC)	DOI: 10.1016/j.annonc.2020.10.024; CENTRAL:CN-02202227	CENTRAL; Embase	IMpassion031	Secondary/companion report of an included study
30	2020	IMpassion031: Results from a phase III study of neoadjuvant (neoadj) atezolizumab + chemotherapy in early triple-negative breast cancer (TNBC)	DOI: 10.1016/j.annonc.2020.08.2239; CENTRAL:CN-02176731	CENTRAL; Embase	IMpassion031	Secondary/companion report of an included study
31	2020	Neoadjuvant Anthracycline Followed by Toripalimab Combined With Nab-paclitaxel in Patients With Early Triple-negative Breast Cancer	Not available in audit record	Embase	—	Trial registration/protocol; companion to NeoTENNIS
32	2020	Predictive markers of response to durvalumab concurrent with nab-paclitaxel and dose dense doxorubicin cyclophosphamide (ddAC) neoadjuvant therapy for triple negative breast cancer (TNBC)	DOI: 10.1158/1538-7445.sabcs19-p3-09-05	Embase	—	Conference/meeting abstract only; no eligible full-text primary article
33	2020	A phase II single-arm, multicenter, open-label neoadjuvant study of pembrolizumab in combination with nab-paclitaxel followed by pembrolizumab in combination with epirubicin and cyclophosphamide in patients with triple-negative breast cancer: Neoimmunoboost	DOI: 10.1200/jco.2020.38.15_suppl.e12647	Embase	NeoImmunoboost	Secondary/companion report of an included study
34	2019	A Phase-2, Two-Cohort Trial of Neoadjuvant Nab-Paclitaxel and Alpelisib in Anthracycline Refractory Triple Negative Breast Cancer With PIK3CA or PTEN Alterations	Not available in audit record	Embase	—	Ineligible regimen: no platinum or immune checkpoint inhibitor
35	2019	Treatment of Triple-negative Breast Cancer With Albumin-bound Paclitaxel as Neoadjuvant Therapy: a Prospective Randomized Controlled Clinical Trial	Not available in audit record	Embase	—	Trial registration/protocol or no eligible full-text results report identified
36	2019	Pathologic response to neoadjuvant therapy with nabpaclitaxel plus carboplatin followed by anthracycline regimen in triple-negative breast cancer	DOI: 10.1200/jco.2019.37.15_suppl.e12117	Embase	—	Conference/meeting abstract only; no eligible full-text primary article
37	2019	BRCA mutations and not type 1 tandem duplicator phenotypes are associated with pathological complete response in patients with triple negative breast cancer undergoing neoadjuvant carboplatin/nab-paclitaxel	DOI: 10.1158/15387445.sabcs18-p3-06-09	Embase	—	Conference/meeting abstract only; no eligible full-text primary article

No.	Year	Report/citation identification	DOI / trial or source identifier	Source(s)	Study family	Specific reason for exclusion
38	2019	KEYNOTE-173: Phase 1b multicohort study of pembrolizumab (Pembro) in combination with chemotherapy as neoadjuvant treatment for triple-negative breast cancer (TNBC)	DOI: 10.1158/15387445.sabcs18-pd5-01; GS:221	Embase; Google Scholar	KEYNOTE-173	Conference abstract/companion report; linked full-text KEYNOTE-173 article used for inclusion
39	2019	Phase II trial of neoadjuvant carboplatin and nab-paclitaxel in patients with locally advanced triple negative breast cancer	DOI: 10.1158/1538-7445.sabcs18-p1-15-07	Embase	Yuan	Secondary/companion report of an included study
40	2018; 2019	Higher efficacy of 2-week nab-paclitaxel compared to solvent-based paclitaxel in neoadjuvant chemotherapy of triple negative breast cancers	DOI: 10.1158/1538-7445.sabcs18-p1-15-23	Embase	—	Conference/meeting abstract only; no eligible full-text primary article
41	2018	Neoadjuvant trial of nab-paclitaxel and atezolizumab (Atezo), a PD-L1 inhibitor, in patients (pts) with chemo-insensitive triple negative breast cancer (TNBC)	DOI: 10.1093/annonc/mdy270.219; GS:96	Embase; Google Scholar	—	Conference/meeting abstract only; companion to Yam atezolizumab study
42	2018	Randomized phase II neoadjuvant study (GeparNuevo) to investigate the addition of durvalumab to a taxane-anthracycline containing chemotherapy in triple negative breast cancer (TNBC)	DOI: 10.1200/jco.2018.36.15_suppl.104; CENTRAL:CN-01791341	CENTRAL; Embase	GeparNuevo	Secondary/companion report of an included study
43	2018	Durvalumab (MEDI4736) concurrent with nab-paclitaxel and dose dense doxorubicin cyclophosphamide (ddAC) as neoadjuvant therapy for triple negative breast cancer (TNBC)	DOI: 10.1200/jco.2018.36.15_suppl.586	Embase	—	Conference/meeting abstract only; no eligible full-text primary article
44	2018	Pathologic response to neoadjuvant therapy with nab-paclitaxel plus carboplatin followed by anthracycline regimen in triple-negative breast cancer	DOI: 10.1200/jco.2018.36.15_suppl.e12660	Embase	—	Conference/meeting abstract only; no eligible full-text primary article
45	2018	IMpassion031: A phase III study comparing neoadjuvant atezolizumab vs placebo in combination with nab-paclitaxel-based chemotherapy in early triple-negative breast cancer (TNBC)	DOI: 10.1158/15387445.sabcs17-ot2-07-03; CENTRAL:CN-01573325	CENTRAL; Embase	IMpassion031	Secondary/companion report of an included study
46	2017	IMpassion031: A phase III study comparing neoadjuvant atezolizumab (atezo) vs placebo in combination with anthracycline/ nab-paclitaxel (nab-pac)-based chemotherapy in early triplenegative breast cancer (eTNBC)	DOI: 10.1093/annonc/mdx362.061; CENTRAL:CN-01439516	CENTRAL; Embase	IMpassion031	Secondary/companion report of an included study
47	2017	A Phase III Randomized Study to Investigate the Efficacy and Safety of Atezolizumab (Anti-PD-L1 Antibody) in Combination With Neoadjuvant Anthracycline/Nab-Paclitaxel-Based Chemotherapy Compared With Placebo and Chemotherapy in Patients With Primary Invasive Triple-Negative Breast Cancer	Not available in audit record	Embase	—	Trial registration/protocol or no eligible full-text results report identified
48	2017	Safety of MEDI4736 (anti-PD-L1 antibody) administered concomitant with weekly nab-paclitaxel and dose dense doxorubicin/cyclophosphamide (ddAC) as neoadjuvant chemotherapy for stage I-III triple negative breast cancer (TNBC): A Phase I/II trial	CENTRAL:CN-01397522	CENTRAL; Embase	—	Conference/meeting abstract only; early safety/companion report
49	2017	Association of molecular subtype, proliferation, and immune genes with efficacy of carboplatin versus gemcitabine addition to taxane-based, anthracycline-free neoadjuvant chemotherapy in early triple-negative breast cancer (TNBC): Results of the randomized WSG ADAPT-TN trial	CENTRAL:CN-01397337	CENTRAL; Embase	WSG-ADAPT-TN	Secondary/companion report of an included study
50	2017	Triple-negative first-line study: Neoadjuvant trial of nab-paclitaxel and atezolizumab, a PD-L1 inhibitor, in patients with triple negative breast cancer (TNBC) (NCT02530489)	DOI: 10.1158/1538-7445.sabcs16-ot2-01-14; NCT:NCT02530489	CENTRAL; Embase	—	Conference/meeting abstract only; no eligible full-text primary article
51	2016	A Phase II One-arm Open-label Neoadjuvant Study of Pembrolizumab in Combination With Nab-paclitaxel Followed by Pembrolizumab in Combination With Epirubicin and Cyclophosphamide in Patients With Triple Negative Breast Cancer	Not available in audit record	Embase	—	Trial registration/protocol or no eligible full-text results report identified
52	2016	Comparison of 12 weeks neoadjuvant Nab-paclitaxel combined with carboplatinum vs. gemcitabine in triple-negative breast cancer: WSG - ADAPT TN randomized phase II trial	DOI: 10.1158/1538-7445.sabcs15-p1-13-01; 10.1158/1538-7445.sabcs15-p1-14-11; 10.1158/1538-7445.sabcs15-s6-07; 10.1159/000444354	Embase	WSG-ADAPT-TN	Secondary/companion report of an included study
53	2016	Phase II trial of neoadjuvant chemotherapy with carboplatin and nab-paclitaxel in patients with triple negative locally advanced and inflammatory breast cancer	DOI: 10.1158/1538-7445.sabcs15-p1-14-10	Embase	Yuan	Secondary/companion report of an included study
54	2015	Neo-Adjuvant Study With the PDL1-directed Antibody in Triple Negative Locally Advanced Breast Cancer Undergoing Treatment With Nab-paclitaxel and Carboplatin	Not available in audit record	Embase	—	Trial registration/protocol or no eligible full-text results report identified
55	2015	Triple-Negative First-Line Study: Neoadjuvant Trial of Nab-Paclitaxel and MPDL3280A, a PDL-1 Inhibitor in Patients With Triple Negative Breast Cancer	Not available in audit record	Embase	—	Trial registration/protocol or no eligible full-text results report identified
56	2015	Single Arm Neoadjuvant Phase I/II Study of MEDI4736 (Anti-PD-L1 Antibody) Concomitant With Weekly Nab-paclitaxel and Dose-dense Doxorubicin/Cyclophosphamide (ddAC) Chemotherapy for Clinical Stage I-III Triple Negative Breast Cancer	Not available in audit record	Embase	—	Trial registration/protocol or no eligible full-text results report identified
57	2015	Efficacy of 12 weeks neoadjuvant nab-paclitaxel combined with carboplatinum vs. gemcitabine in triple-negative breast cancer: WSG-ADAPT TN randomized phase II trial	Not available in audit record	Embase	WSG-ADAPT-TN	Secondary/companion report of an included study
58	2012	Neoadjuvant weekly nab-paclitaxel (wA), carboplatin (Cb) plus bevacizumab (B) with or without dose-dense doxorubicin-cyclophosphamide (ddAC) plus B in ER+/HER2-negative (HR+) and triple-negative (TN) breast cancer (BrCa): A BrUOG study	Not available in audit record	Embase	—	Ineligible/companion antiangiogenic regimen report; does not add an independent eligible study
59	2012	Neoadjuvant bevacizumab with weekly nab-paclitaxel plus carboplatin followed by doxorubicin plus cyclophosphamide (AC) for triple-negative breast cancer	Not available in audit record	Embase	—	Ineligible/companion antiangiogenic regimen report; does not add an independent eligible study
60	2012	Phase II Trial of Neoadjuvant Chemotherapy With Carboplatin and NAB-Paclitaxel in Patients With Locally Advanced and Inflammatory Triple Negative Breast Cancer	Not available in audit record	Embase	Yuan	Secondary/companion report of an included study

No.	Year	Report/citation identification	DOI / trial or source identifier	Source(s)	Study family	Specific reason for exclusion
61	2010	Neoadjuvant bevacizumab with weekly nanoparticle albumin bound nab-paclitaxel plus carboplatin followed by doxorubicin plus cyclophosphamide (AC) for triple-negative breast cancer	Not available in audit record	Embase	—	Ineligible/companion antiangiogenic regimen report; does not add an independent eligible study
62	2008	Neoadjuvant Weekly Nab-paclitaxel (Abraxane®) Plus Carboplatin Followed By Doxorubicin Plus Cyclophosphamide With Bevacizumab Added Concurrently To Chemotherapy For Palpable And Operable Triple Negative Invasive Breast Cancer	Not available in audit record	Embase	—	Ineligible/companion antiangiogenic regimen report; does not add an independent eligible study
63	2019	Nab-paclitaxel Based Regimens VS Paclitaxel Based Regimens in Neoadjuvant Treatment for TNBC	NCT:NCT04067102	CENTRAL	—	Withdrawn trial registration; no results
64	2023	Stereotactic Radiotherapy Combined With Adebrelimab and TCb (Nab-paclitaxel + Carboplatin) in Neoadjuvant Treatment of TNBC	NCT:NCT06165900	CENTRAL	Chen / NCT05132790	Secondary/companion report of an included study
65	2017	Pilot study to assess physical exercise intervention in neoadjuvant treatment with nab-paclitaxel in patients with triple negative breast cáncer	CENTRAL:CN-01890754	CENTRAL	—	Ancillary intervention study; not an eligible neoadjuvant treatment efficacy report
66	2017	A Study of Atezolizumab with Neoadjuvant Anthracycline/NAB Paclitaxel Based Chemotherapy Compared with Placebo and Chemotherapy in Patients with Primary Invasive Triple-Negative Breast Cancer	CENTRAL:CN-01886489	CENTRAL	—	Trial registration/protocol or no eligible full-text results report identified
67	2025	Neoadjuvant chemotherapy plus camrelizumab vs. chemotherapy in patients with locally advanced immunomodulatory triple-negative breast cancer: a prospective, randomized, open-label, phase 2 trial Available	CENTRAL:CN-02968972	CENTRAL	—	Secondary/duplicate source record of CamRelief primary report
68	2017	A randomized phase II neoadjuvant study (GeparNuevo) to investigate the addition of durvalumab, a PD-L1 antibody, to a taxane-anthracycline containing chemotherapy in triple negative breast cancer (TNBC)	CENTRAL:CN-01397361	CENTRAL	GeparNuevo	Secondary/companion report of an included study
69	2019	Albumin-bound paclitaxel combined with carboplatin versus epirubicin combined with docetaxel as neoadjuvant therapy for triple-negative breast cancer: a multicenter randomized controlled phase IV clinical trial	CENTRAL:CN-02065223	CENTRAL	—	Wrong taxane/intervention; no nab-paclitaxel
70	2020	A prospective, open, multicenter, randomized controlled trial for the effect of albumin-bound paclitaxel combined with cisplatin versus epirubicin combined with cyclophosphamide sequential docetaxel neoadjuvant therapy for triple negative breast cancer	CENTRAL:CN-02239108	CENTRAL	—	Wrong taxane/intervention; no nab-paclitaxel
71	2016	Neo-Adjuvant study with the PDL1-directed antibody in Triple Negative, Early High-Risk and Locally Advanced Breast Cancer undergoing treatment with nab-paclitaxel and carboplatin. NeoTRIPaPDL1 (Neoadjuvant therapy in TRIPle negative breast cancer with antiPDL1)	CENTRAL:CN-01854346	CENTRAL	NeoTRIPaPDL1	Secondary/companion report of an included study
72	2024	Neoadjuvant anthracycline followed by toripalimab combined with nab-paclitaxel in patients with early triple-negative breast cancer (NeoTENNIS): a single-arm …	GS:12	Google Scholar	NeoTENNIS	Secondary/companion report of an included study
73	2025	… and exploratory analysis of neoadjuvant tislelizumab (a PD-1 inhibitor) plus nab-paclitaxel followed by epirubicin/cyclophosphamide for triple-negative breast cancer …	GS:13	Google Scholar	—	Secondary/duplicate source record of TREND primary report
74	2022	… controlled study of neoadjuvant atezolizumab or placebo, combined with nab-paclitaxel and anthracycline-based chemotherapy in early triple-negative breast cancer …	GS:34	Google Scholar	—	Subgroup/secondary report of IMpassion031
75	2018; 2020	… (MEDI4736) concurrent with nab-paclitaxel and dose dense doxorubicin cyclophosphamide (ddAC) as neoadjuvant therapy for triple negative breast cancer (TNBC).	GS:35; GS:94	Google Scholar	—	Secondary/companion report of Foldi/durvalumab study
76	2021	Neoadjuvant atezolizumab (atezo) and nab-paclitaxel (nab-p) in patients (pts) with triple-negative breast cancer (TNBC) with suboptimal clinical response to …	GS:38	Google Scholar	—	Secondary/companion report of Yam atezolizumab study
77	2017	… neoadjuvant atezolizumab (atezo) vs placebo in combination with anthracycline/nab-paclitaxel (nab-pac)–based chemotherapy in early triple-negative breast cancer …	GS:87	Google Scholar	—	Trial design/companion report of IMpassion031
78	2021	… phase II trial of anthracycline-free and anthracycline-containing neoadjuvant carboplatin chemotherapy regimens in stage I–III triple-negative breast cancer …	GS:103	Google Scholar	—	Wrong intervention; no nab-paclitaxel-containing regimen
79	2017	… nab-paclitaxel and dose dense doxorubicin/cyclophosphamide (ddAC) as neoadjuvant chemotherapy for stage I-III triple negative breast cancer (TNBC): a phase I/II …	GS:106	Google Scholar	—	Conference/meeting abstract only; companion to Foldi/durvalumab
80	2018	… : A phase III study comparing neoadjuvant atezolizumab vs placebo in combination with nab-paclitaxel–based chemotherapy in early triple-negative breast cancer …	GS:116	Google Scholar	—	Trial design/companion report of IMpassion031
81	2016	Abstract S6-07: comparison of 12 weeks neoadjuvant Nab-paclitaxel combined with carboplatinum vs. gemcitabine in triple-negative breast cancer: WSG-ADAPT TN …	GS:117	Google Scholar	WSG-ADAPT-TN	Secondary/companion report of an included study
82	2023	… response (pCR) to neoadjuvant treatment (NAT) with carboplatin, nab-paclitaxel and anthracycline in early and locally advanced triple-negative breast cancer (TNBC) …	GS:121	Google Scholar	—	Retrospective study and conference abstract only
83	2015	Efficacy of 12 weeks neoadjuvant nab-paclitaxel combined with carboplatinum vs. gemcitabine in triple-negative breast cancer: WSG-ADAPT TN randomized phase II …	GS:122	Google Scholar	WSG-ADAPT-TN	Secondary/companion report of an included study
84	2019	Abstract P1-15-07: Phase II trial of neoadjuvant carboplatin and nab-paclitaxel in patients with locally advanced triple negative breast cancer	GS:133	Google Scholar	—	Conference/meeting abstract only; no eligible full-text primary article
85	2020	LBA11 IMpassion031: results from a phase III study of neoadjuvant (neoadj) atezolizumab+ chemotherapy in early triple-negative breast cancer (TNBC)	GS:134	Google Scholar	IMpassion031	Secondary/companion report of an included study

No.	Year	Report/citation identification	DOI / trial or source identifier	Source(s)	Study family	Specific reason for exclusion
86	2024	… Camrelizumab in Combination with Nab-Paclitaxel and Carboplatin versus Nab-Paclitaxel and Carboplatin as Neoadjuvant Therapy for Triple Negative Breast Cancer …	GS:137	Google Scholar	—	Conference/meeting abstract only; no eligible full-text primary article
87	2025	Abstract P5-09-16: Real world experience with Carboplatin plus Nab-paclitaxel as neoadjuvant therapy in patients with early triple negative breast cancer	GS:149	Google Scholar	—	Retrospective/real-world study; prospective design criterion not met
88	2023	… OT2-18-01: Nab-paclitaxel followed by dose-dense epirubicin/cyclophosphamide in neoadjuvant chemotherapy for triple-negative breast cancer: an open-label …	GS:156	Google Scholar	—	Conference/meeting abstract only; no eligible full-text primary article
89	2024	Abstract PO2-03-05: Neoadjuvant anthracycline followed by Toripalimab combined with nab-paclitaxel in patients with stage IIA-IIIC triple negative breast cancer …	GS:160	Google Scholar	—	Conference/meeting abstract only; no eligible full-text primary article
90	2020	… response to durvalumab concurrent with nab-paclitaxel and dose dense doxorubicin cyclophosphamide (ddAC) neoadjuvant therapy for triple negative breast cancer …	GS:166	Google Scholar	—	Secondary biomarker/companion report of Foldi/durvalumab study
91	2021	179P Neoadjuvant camrelizumab plus nab-paclitaxel and epirubicin for early triple-negative breast cancer: A single-arm, open-label, phase II study	GS:210	Google Scholar	—	Conference/meeting abstract only; no eligible full-text primary article
92	2022	170P Phase II study of camrelizumab plus chemotherapy as neoadjuvant therapy in patients with early triple-negative breast cancer	GS:247	Google Scholar	—	Conference/meeting abstract only; no eligible full-text primary article

Abbreviations: pCR, pathological complete response; TNBC, triple-negative breast cancer. The detailed row-level reason is the operative exclusion decision; the summary categories above are only a condensed presentation for readability.

PRISMA 2020 Checklist- Supplementary fileS2

Section and Topic	Item #	Checklist item	Location where item is reported
**TITLE**			
Title	1	Identify the report as a systematic review.	p. 1, Title
**ABSTRACT**			
Abstract	2	See the PRISMA 2020 for Abstracts checklist.	p. 2, Abstract
**INTRODUCTION**			
Rationale	3	Describe the rationale for the review in the context of existing knowledge.	pp. 3-4, Introduction
Objectives	4	Provide an explicit statement of the objective(s) or question(s) the review addresses.	p. 4, Introduction, final paragraph
**METHODS**			
Eligibility criteria	5	Specify the inclusion and exclusion criteria for the review and how studies were grouped for the syntheses.	p. 5, Sec. 2.3
Information sources	6	Specify all databases, registers, websites, organisations, reference lists and other sources searched or consulted to identify studies. Specify the date when each source was last searched or consulted.	p. 5, Sec. 2.2
Search strategy	7	Present the full search strategies for all databases, registers and websites, including any filters and limits used.	p. 5, Sec. 2.2
Selection process	8	Specify the methods used to decide whether a study met the inclusion criteria of the review, including how many reviewers screened each record and each report retrieved, whether they worked independently, and if applicable, details of automation tools used in the process.	pp. 5-6, Sec. 2.4; 2.5, Fig. 1.
Data collection process	9	Specify the methods used to collect data from reports, including how many reviewers collected data from each report, whether they worked independently, any processes for obtaining or confirming data from study investigators, and if applicable, details of automation tools used in the process.	p. 7, Sec. 2.5
Data items	10a	List and define all outcomes for which data were sought. Specify whether all results that were compatible with each outcome domain in each study were sought (e.g. for all measures, time points, analyses), and if not, the methods used to decide which results to collect.	p. 7, Sec. 2.6
	10b	List and define all other variables for which data were sought (e.g. participant and intervention characteristics, funding sources). Describe any assumptions made about any missing or unclear information.	p. 7, Sec. 2.5; Sec. 2.6
Study risk of bias assessment	11	Specify the methods used to assess risk of bias in the included studies, including details of the tool(s) used, how many reviewers assessed each study and whether they worked independently, and if applicable, details of automation tools used in the process.	pp. 7-8, Sec. 2.7
Effect measures	12	Specify for each outcome the effect measure(s) (e.g. risk ratio, mean difference) used in the synthesis or presentation of results.	pp. 7-8, Secs. 2.6 and 2.8

Section and Topic	Item #	Checklist item	Location where item is reported
Synthesis methods	13a	Describe the processes used to decide which studies were eligible for each synthesis (e.g. tabulating the study intervention characteristics and comparing against the planned groups for each synthesis (item #5)).	pp. 7-8, Secs. 2.6 and 2.8
	13b	Describe any methods required to prepare the data for presentation or synthesis, such as handling of missing summary statistics, or data conversions.	pp. 7-9, Secs. 2.6 and 2.8
	13c	Describe any methods used to tabulate or visually display results of individual studies and syntheses.	pp. 9-25, Secs. 3.1-3.4; Tables 1-6; Figs. 1-3
	13d	Describe any methods used to synthesize results and provide a rationale for the choice(s). If meta-analysis was performed, describe the model(s), method(s) to identify the presence and extent of statistical heterogeneity, and software package(s) used.	pp. 8-9, Sec. 2.8.
	13e	Describe any methods used to explore possible causes of heterogeneity among study results (e.g. subgroup analysis, meta-regression).	pp. 8-9, Sec. 2.8;
	13f	Describe any sensitivity analyses conducted to assess robustness of the synthesized results.	p. 9, Sec. 2.8
Reporting bias assessment	14	Describe any methods used to assess risk of bias due to missing results in a synthesis (arising from reporting biases).	p. 9, Sec. 2.8
Certainty assessment	15	Describe any methods used to assess certainty (or confidence) in the body of evidence for an outcome.	pp. 7-8, Sec. 2.7
**RESULTS**			
Study selection	16a	Describe the results of the search and selection process, from the number of records identified in the search to the number of studies included in the review, ideally using a flow diagram.	pp. 5-6, 9-10; Secs. 2.4 and 3.1; Fig. 1
	16b	Cite studies that might appear to meet the inclusion criteria, but which were excluded, and explain why they were excluded.	p. 6, Fig. 1
Study characteristics	17	Cite each included study and present its characteristics.	pp. 10-14, Sec. 3.2; Table 1
Risk of bias in studies	18	Present assessments of risk of bias for each included study.	pp. 14-16, Sec. 3.3; Tables 2A-2C
Results of individual studies	19	For all outcomes, present, for each study: (a) summary statistics for each group (where appropriate) and (b) an effect estimate and its precision (e.g. confidence/credible interval), ideally using structured tables or plots.	pp. 17-24, Sec. 3.4; Tables 3-5A; Figs. 2-3
Results of syntheses	20a	For each synthesis, briefly summarise the characteristics and risk of bias among contributing studies.	pp. 10-16, Secs. 3.2-3.3; Tables 1-2C
	20b	Present results of all statistical syntheses conducted. If meta-analysis was done, present for each the summary estimate and its precision (e.g. confidence/credible interval) and measures of statistical heterogeneity. If comparing groups, describe the direction of the effect.	pp. 21-25, Sec. 3.4; Tables 5-6; Figs. 2-3

Section and Topic	Item #	Checklist item	Location where item is reported
	20c	Present results of all investigations of possible causes of heterogeneity among study results.	pp. 22-24, Sec. 3.4;
	20d	Present results of all sensitivity analyses conducted to assess the robustness of the synthesized results.	pp. 22-24, Sec. 3.4; Table 5A
Reporting biases	21	Present assessments of risk of bias due to missing results (arising from reporting biases) for each synthesis assessed.	p. 25, Sec. 3.4; formal publication-bias assessment not undertaken (4 RCTs).
Certainty of evidence	22	Present assessments of certainty (or confidence) in the body of evidence for each outcome assessed.	pp. 14-16, Sec. 3.3; Table 2C
**DISCUSSION**			
Discussion	23a	Provide a general interpretation of the results in the context of other evidence.	pp. 25-27, Discussion
	23b	Discuss any limitations of the evidence included in the review.	p. 27, Discussion, limitations paragraph
	23c	Discuss any limitations of the review processes used.	p. 27, Discussion.
	23d	Discuss implications of the results for practice, policy, and future research.	pp. 26-28, Discussion and Conclusions
**OTHER INFORMATION**			
Registration and protocol	24a	Provide registration information for the review, including register name and registration number, or state that the review was not registered.	pp. 4-5, Sec. 2.1; PROSPERO CRD420261418350
	24b	Indicate where the review protocol can be accessed, or state that a protocol was not prepared.	p. 5, Sec. 2.1 location of a separate full protocol is not stated.
	24c	Describe and explain any amendments to information provided at registration or in the protocol.	pp. 4-5, Sec. 2.1
Support	25	Describe sources of financial or non-financial support for the review, and the role of the funders or sponsors in the review.	p. 28, Funding

Section and Topic	Item #	Checklist item	Location where item is reported
Competing interests	26	Declare any competing interests of review authors.	p. 28, Conflicts of Interest
Availability of data, code and other materials	27	Report which of the following are publicly available and where they can be found: template data collection forms; data extracted from included studies; data used for all analyses; analytic code; any other materials used in the review.	p. 28, Data Availability Statement; study-level dataset available from corresponding author on reasonable request.

From: Page MJ, McKenzie JE, Bossuyt PM, Boutron I, Hoffmann TC, Mulrow CD, et al. The PRISMA 2020 statement: an updated guideline for reporting systematic reviews. BMJ 2021;372:n71. doi: 10.1136/bmj.n71. This work is licensed under CC BY 4.0. To view a copy of this license, visit https://creativecommons.org/licenses/by/4.0/
